# Thalattosauria in time and space: a review of thalattosaur spatiotemporal occurrences, presumed evolutionary relationships and current ecological hypotheses

**DOI:** 10.1186/s13358-024-00333-6

**Published:** 2024-09-26

**Authors:** Dylan Bastiaans

**Affiliations:** 1 Paläontologisches Institut, Karl-Schmid-Strasse 4, 8006 Zurich, Switzerland; 2 Natural History Museum Maastricht, Centre Céramique, De Bosquetplein 7, 6211 KJ Maastricht, The Netherlands

**Keywords:** Triassic, Thalattosauriformes, Marine reptiles, Monte San Giorgio, Biogeography, Ecology, Phylogeny

## Abstract

In the wake of the greatest mass extinction in Earth’s history, the End-Permian Mass Extinction, the Triassic was a time of recovery and innovation. Aided by warm climatic conditions and favorable ecological circumstances, many reptilian clades originated and rapidly diversified during this time. This set the stage for numerous independent invasions of the marine realm by several reptilian clades, such as Ichthyosauriformes and Sauropterygia, shaping the oceanic ecosystems for the entire Mesozoic. Although comparatively less speciose, and temporally and latitudinally more restricted, another marine reptile clade, the Thalattosauriformes, stands out because of their unusual and highly disparate cranial, dental and skeletal morphology. Research on Thalattosauriformes has been hampered by a historic dearth of material, with the exception of rare material from Lagerstätten and highly fossiliferous localities, such as that from the UNESCO world heritage site of Monte San Giorgio. Consequently, their evolutionary origins and paleobiology remain poorly understood. The recent influx of new material from southwestern China and North America has renewed interest in this enigmatic group prompting the need for a detailed review of historic work and current views. The earliest representatives of the group may have been present from the late Early Triassic onwards in British Columbia. By the Ladinian the group had achieved a wide distribution across the northern hemisphere, spanning the eastern Panthalassic as well as the eastern and western Tethyan provinces. Distinct morphological and likely ecological differences exist between the two major clades of Thalattosauriformes, the Askeptosauroidea and the Thalattosauroidea, with the latter showing a higher degree of cranial and skeletal morphological disparity. In-group relationships remain poorly resolved beyond this bipartition. Overall, thalattosaurs may be closely related to other marine reptile groups such as ichthyopterygians and sauropterygians. However, their exact position within Diapsida remains elusive. Future focal points should utilize modern digital paleontological approaches to explore the many fragmentary specimens of otherwise poorly sampled localities.

## Introduction

In the wake of the greatest mass extinction in earth’s history (i.e. End-Permian Mass Extinction Event, EPME, ~ 251.9 million years ago), the Triassic was a time of recovery, innovation and radiation for many important extinct and extant vertebrate and invertebrate lineages (Benton, 2005, 2014, 2016, 2018; Benton & Chen, 2012; Benton et al., 2013; Kelley et al., 2014; Renesto & Dalla Vecchia, 2017; Liu & Sander, 2019; Moon & Stubbs, 2020; Corso et al., 2022; Kear et al., 2023; Cawthorne et al., 2024). The environmental perturbations of the EPME were reflected in biotic crises that affected both the terrestrial and marine ecosystems, albeit not concurrently nor to the same severity (Benton, 2016, 2018; Benton & Twitchett, 2003; Corso et al., 2022; Galasso et al., 2022; Nowak et al., 2019, 2020; Widmann et al., 2020 and references therein). Extinction was taxonomically, ecologically, physiologically and spatially selective (Dal Corso et al., 2022).

The severe multi-phased extinctions in the terrestrial and marine realms resulted in a complete restructuring of ecosystems, both taxonomically and trophically, and consequently the development of new ecological niches (Benton, 2016; Benton et al., 2013; Cawthorne et al., 2024; Chen & Benton, 2012; Corso et al., 2022; Kelley, 2012). Recovery after the EPME is highly differentiated in timing, speed, mode and spatiotemporal spread for various vertebrate and invertebrate lineages (Bardet, 1994; Brayard et al., 2009; Song et al., 2011, 2018; Chen & Benton, 2012; Benton et al., 2013; Benton, 2014, 2016, 2018; Kelley et al., 2014; Scheyer et al., 2014; Friedman, 2015; Hofmann et al., 2015; Kelley & Pyenson, 2015; Liu & Sander, 2019; Mays et al., 2020; Moon & Stubbs, 2020; Sun et al., 2020; Dal Corso et al., 2022; Galasso et al., 2022; Du et al., 2023; Jiang et al., 2023).

The warm climatic conditions created favorable circumstances for many reptilian clades to explore new environments and new ecological opportunities (i.e. unexplored niche space). This provided the setting for numerous independent invasions of the marine realm by various reptilian clades (Bardet, 1994; Bardet et al., 2014; Benson & Butler, 2011; Benton, 2014; Moon & Stubbs, 2020; Motani, 2009). Marine reptiles attain their peak diversity during the Triassic, emphasizing the importance of this period for understanding broader patterns of reptilian evolution (Moon & Stubbs, 2020; Motani, 2009).

The various marine tetrapod groups went on to fill a diverse set of ecological niches that are currently largely represented by extant marine amniotes, including apex predator roles in many Triassic ecosystems (Benton, 2005; Thorne et al., 2011; Kelley, 2012; Benton et al., 2013; Bardet et al., 2014; Kelley et al., 2014; Kelley & Pyenson, 2015; Renesto & Dalla Vecchia, 2017; Sun et al., 2020). Predatory amphibians and fish which constituted important predators in Permian coastal and marine ecosystems, respectively, were replaced by marine reptiles such as the taxonomically and spatiotemporally highly successful Ichthyosauriformes and Sauropterygia (Kear et al., 2023; Scheyer et al., 2014). Additional sparse marine invasions during the Triassic by reptilian clades gave rise to groups such as: protorosaurs and other aquatic archosauromorphs, hupehsuchians, and saurosphargids (Bardet et al., 2014; Kelley, 2012; Motani, 2009). Together all these clades comprise a polyphyletic assemblage collectively termed ‘Mesozoic marine reptiles’ (Bardet, 1994; Kelley, 2012). These groups exhibit a wide array of diets and swimming styles previously unexplored by tetrapods in the Palaeozoic (Bardet et al., 2014; Moon & Stubbs, 2020; Motani, 2009). Their presence within two million years after the EPME and subsequent rapid radiation represents one of the most significant events in Mesozoic vertebrate evolutionary history (Kelley, 2012; Kelley et al., 2014; Scheyer et al., 2014; Renesto & Dalla Vecchia, 2017; Moon & Stubbs, 2020; Wang et al., 2022; Jiang et al., 2023).

Many of these newly emerging marine clades explored unique trophic guilds and ecological niches not represented in the Permian oceans (Bardet et al., 2014; Benton, 2014; Benton et al., 2013). Of these marine reptile clades, the Thalattosauriformes (from here on “thalattosaurs”) represent one of the most enigmatic. Despite being known for over a century, our understanding of their evolution and paleobiology is still limited. This review aims to summarize current knowledge to provide a foundation for future work.

### Thalattosauriformes

Thalattosauriformes (sensu Nicholls, 1999) is an exclusively Triassic clade of secondarily aquatic reptiles (Benton, 2014; Renesto & Dalla Vecchia, 2017). Despite the relatively modest evolutionary timespan, possibly ranging from the late Early to Late Triassic (< 50 million years), this group is characterized by exceptional morphological disparity, particularly in their body sizes (1– > 4 m), rostral shapes and dentition types, likely reflecting different lifestyles and feeding habits (Bardet et al., 2014; Bastiaans et al., 2023; Benton, 2014; Motani, 2009; Naish, 2023; Rieppel, 2019; Rieppel et al., 2005). Thalattosaurs are moderately speciose, with currently 14–16 recognized genera and 18–24 recognized species, and display a cosmopolitan or wide distribution across the low latitude coastal to coastal-pelagic environments in the northern hemisphere (Merriam, 1905; Nopcsa, 1925; Peyer, 1936a, 1936b; Kuhn, 1952; Nicholls & Brinkman, 1993; Nicholls, 1999; Rieppel et al., 2000; Yin et al., 2000; Müller, 2005, 2007; Druckenmiller et al., 2020; Bastiaans et al., 2023).

Thalattosauriformes are slender with proportionally long, narrow bodies, elongated tails and plesiopedal limbs, all features that are indicative of a plesiomorphic axial undulatory swimming style. This likely allowed them to achieve rapid bursts of acceleration but made them incapable of sustaining long distance swimming at speed (Müller, 2002; Benson & Butler, 2011; Kelley et al., 2014; Sun et al., 2020). Other aquatic adaptations include tall and laterally compressed tails, streamlined crania and retracted external nares placed just in front of the orbit (Benton, 2005, 2014; Druckenmiller et al., 2020; Müller, 2002; Rieppel et al., 2005).

**Fig. 1:**
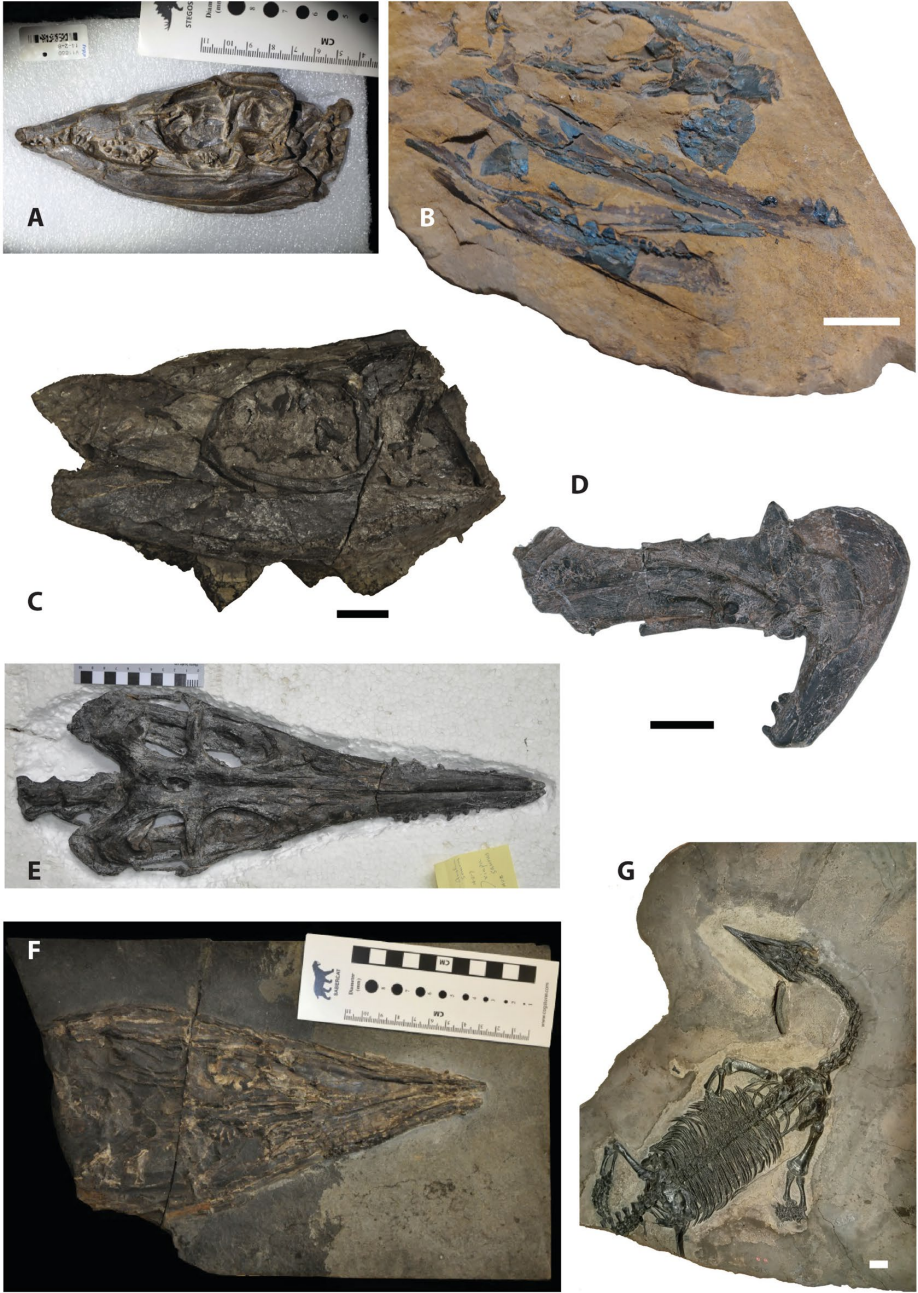
Fossil remains of various thalattosaur taxa **A** *Xinpusaurus suni* (IVPP V 11860)*. **B** *Paralonectes merriami* (holotype, TMP 89.127.1). **C** *Thalattosaurus alexandrae* (USNM 10926) *. **D** XNGM WS-22-R5 *. **E** *Anshunsaurus huangguoshuensis* (IVPP V 11835) *. **F** *Askeptosaurus italicus* (PIMUZ T 5392) *. **G** *Endennasaurus acutirostris* (MBSN 5170) ***. *courtesy of T.M. Scheyer; **courtesy of Jun Chai and Da-Yong Jiang; ***courtesy of Annalisa Aiello. Scale bars **B**, **C**, **D**, **G** equal 2 cm

Some taxa possess straight, tooth-bearing or edentulous rostra (e.g. *Anshunsaurus, Askeptosaurus, Endennasaurus*), whereas others exhibit tapering pointed snouts (e.g. *Xinpusaurus*) or even moderately to strongly ventrally deflecting anterior rostra (e.g. *Clarazia schinzi*, *Thalattosaurus alexandrae, Hescheleria ruebeli, Nectosaurus halius*) (Fig. 1). Thalattosaurs show a remarkable degree of dental disparity and variation in dental coverage, including extreme heterodonty and dentigerous or edentulous palatal elements, or complete edentulism. Thalattosaurs such as *Xinpusaurus* and *Thalattosaurus* display conical or spatulate teeth and globular, bulbous or blunted teeth (Benton et al., 2013; Li et al., 2016; Motani, 2009; Müller, 2005; Stubbs & Benton, 2016). *Askeptosaurus* and *Anshunsaurus* on the other hand are characterized by homodont dentitions and an edentulous palatal region (Benton et al., 2013; Li et al., 2016; Motani, 2009; Müller, 2005; Stubbs & Benton, 2016). Neck length also varies in thalattosaurs with some taxa having long necks with > 10 cervical vertebrae (e.g. *Askeptosaurus*), while others having as few as four cervical vertebrae (e.g. *Concavispina*) (Cheng et al., 2007b; Liu et al., 2013; Müller, 2002, 2005; Zhao et al., 2013).

Thalattosaurs further strongly differ from the (derived) diapsid skull configuration of other Mesozoic marine reptiles, in that they show considerable reduction in the size of the upper temporal fenestra (i.e. slit-like opening or completely closed) (Fig. 1E). In addition, they show posterodorsally placed external nares, elongated premaxillary rostra, large orbits, long jaws; and retain a large ventrolateral emargination with an open lower temporal arcade (Benton, 2014; Druckenmiller et al., 2020; Kuhn-Schnyder, 1988; Motani, 2009; Müller, 2005; Naish, 2023; Nicholls, 1999; Rieppel, 2019). This cranial morphology is a stark contrast to the condition observed in for instance nothosauroid sauropterygians, where a trend towards progressively enlarged and elongated upper temporal fenestrations can be observed, which may reflect specialized feeding mechanics (Rieppel, 2002). Furthermore, thalattosaurs display deeply emarginated posterior skull tables and anteriorly displaced occiput (Fig. 1E) that may have accommodated powerful neck musculature (Müller, 2002; Naish, 2023; Nicholls, 1999; Rieppel, 1987, 2019).

Thalattosaurs are often described as slender anguilliform ambush predators, that were mainly propelled forward using their exceptionally elongated, laterally compressed tail, while their short and robust limbs may have allowed for some terrestrial locomotion (Bardet et al., 2014; Benton, 2014; Merriam, 1905; Motani, 2009; Müller, 2005; Nicholls, 1999; Rieppel, 2019; Rieppel et al., 2000). The latter is further illustrated by their manus and pes which are sometimes adorned with sharp claw-like terminal phalanges (Kuhn-Schnyder, 1988). The degree to which thalattosaurs made short incursions into the pelagic zone is currently unknown.

### An enigmatic clade with a problematic fossil record

Much of the historic fossil record of thalattosaurs consists of isolated or fragmentary remains (e.g. Storrs, 1991b; Dalla Vecchia, 1993; Sander et al., 1994; Rieppel & Hagdorn, 1998; Renesto, 2005; Muller, 2007) and finds on scree slopes without stratigraphic context (e.g. Nicholls & Brinkman, 1993) from Triassic shallow marine deposits in North America and Europe. The more complete fossil remains from these locations, often including fully articulated skeletons (Fig. 1B, F, G), are however preserved as highly compacted and sometimes severely weathered slab specimens (Bastiaans et al., 2023; Brinkman, 1988; Druckenmiller et al., 2020; Müller, 2002, 2005; Müller et al., 2005; Nicholls & Brinkman, 1993; Peyer, 1936a, 1936b; Rieppel, 2019). This significantly limits the morphological detail and information that can be gained from studying these remains and has resulted in the clade having been heavily understudied relative to other marine reptile groups.

The Triassic fossil record itself is plagued by various (mega)biases related to geology, taphonomy and sampling, such as the Lagerstätten effect (Bardet, 1994; Benson et al., 2010; Benson & Butler, 2011; Benton et al., 2013; Bardet et al., 2014; Kelley et al., 2014; Liu & Sander, 2019; Du et al., 2023; Woolley et al., 2024). The Triassic marine reptile record is partially influenced by the quantity of fossiliferous deposits, the sampled facies and eustatic sea level changes, resulting in overrepresentation of certain time bins or taxa in phylogenetic analyses (Benson & Butler, 2011; Benson et al., 2010; Woolley et al., 2024). These aspects are likely especially important for the Anisian and Carnian thalattosaur record as well as rich fossiliferous units that historically would not fit the definition of a Lagerstätte (Woolley et al., 2024), such as for instance the Hosselkus Limestone of California and the Sulphur Mountain Formation of British Columbia.

Stratigraphic biases and a general incompleteness of the fossil record for marine reptiles during this period, as indicated by extensive ghost lineages for many clades, also heavily influence diversity estimates (Thorne et al., 2011; Kelley et al., 2014). Comparatively few locations preserve the Early Triassic, Norian and Rhaetian shallow to moderately deep marine settings likely inhabited by thalattosaurs, making these extremely prone to underestimation of diversity and disparity (Bardet et al., 2014). “Phylogenetic Lagerstätten effect” (Woolley et al., 2024) may also be particularly important for thalattosaur research as their fossil record largely consists of fragmentary isolated discoveries, with few exceptionally well-preserved taxa representing the bulk of morphological information. 

Collection and historical bias emphasized work on thalattosaur-bearing Anisian-Ladinian and Carnian deposits, respectively represented by much of western Europe, western USA and only recently China. Exploration of Lower Triassic sediments in remote locations such as those of British Columbia are quite a logistic undertaking and therefore heavily underrepresented.

Biological biases may also influence analyses on thalattosaurs as top trophic levels are represented by fewer specimens in their respective ecosystem, making them inherently even less likely to fossilize and be discovered (Bardet et al., 2014). Lastly, neither the first nor last occurrence of many taxa can be detailed as these periods of low population sizes and presumed low diversity are extremely susceptible to these various biases (Bardet et al., 2014).

During the Early Triassic, the marine reptile fossil record is largely composed of fragmentary remains with a seemingly low taxic diversity for most clades (Jiang et al., 2023). This pattern may not be genuine as the poor quality of their remains have precluded definite historic determination (e.g. Bastiaans et al., 2023; Brinkman, 1988; Nicholls & Brinkman, 1993), and many Lower Triassic localities may represent the wrong environmental setting for early representatives of many clades. Important fossil localities for marine reptiles of this age can be found in the United States (Nevada), Canada (British Columbia), Svalbard, Japan and China (Yuan’an and Chaohu), almost all of which represent moderate to deep marine settings with predominantly pelagically-adapted ichthyosaurs (Callaway & Brinkman, 1989; Du et al., 2023; Liu & Sander, 2019). The shallow intraplatform basin of Yuan’an (Liu & Sander, 2019) may be a suitable setting for thalattosaurs, however, to date no remains have been discovered there. The Anisian-Ladinian deposits of Nevada (Fossil Hill Member), Monte San Giorgio (Besano Formation, Switzerland and Italy), and China (Guanling Formation in Panxian and Luoping) preserved moderately deep shelf deposits and intraplatform basins with periodic connectivity to the open sea. These environments were full of ichthyosaurs (Nevada) and shallow marine vertebrates such as fish and diapsid marine reptiles (Benton et al., 2013; Liu & Sander, 2019; Müller, 2005). Of these only the Besano Formation has produced few, but relatively complete thalattosaur remains thus far. Only a single late Ladinian Lagerstätte is known worldwide, the Xingyi Fauna which has produced an abundance of thalattosaur remains (e.g. Chai et al., 2020a, 2020b; Cheng et al., 2007a, 2011; Li et al., 2016; Lu et al., 2018). The Upper Triassic has several famous, but mostly conservation (Konservat), Lagerstätten and rich fossiliferous localities but few for shallow marine reptiles, most famous of which are the Carnian Guanling biota and the Norian-Rhaetian Kössen Formation (Swiss Alps), with additional important localities in British Columbia (Pardonet Formation) and Italy (Sander et al., 2021b).

When compared to small eosauropterygians, which likely had somewhat similar ecological and habitat preferences and sometimes occur in the same rock units, thalattosaur fossils are nonetheless distinctly less abundant. This may hint at a genuine signal of rarity and potentially lower standing diversity, or diversification rates compared to coeval sauropterygians and ichthyosaurs.

### History of thalattosaur discoveries and research

**Fig. 2:**
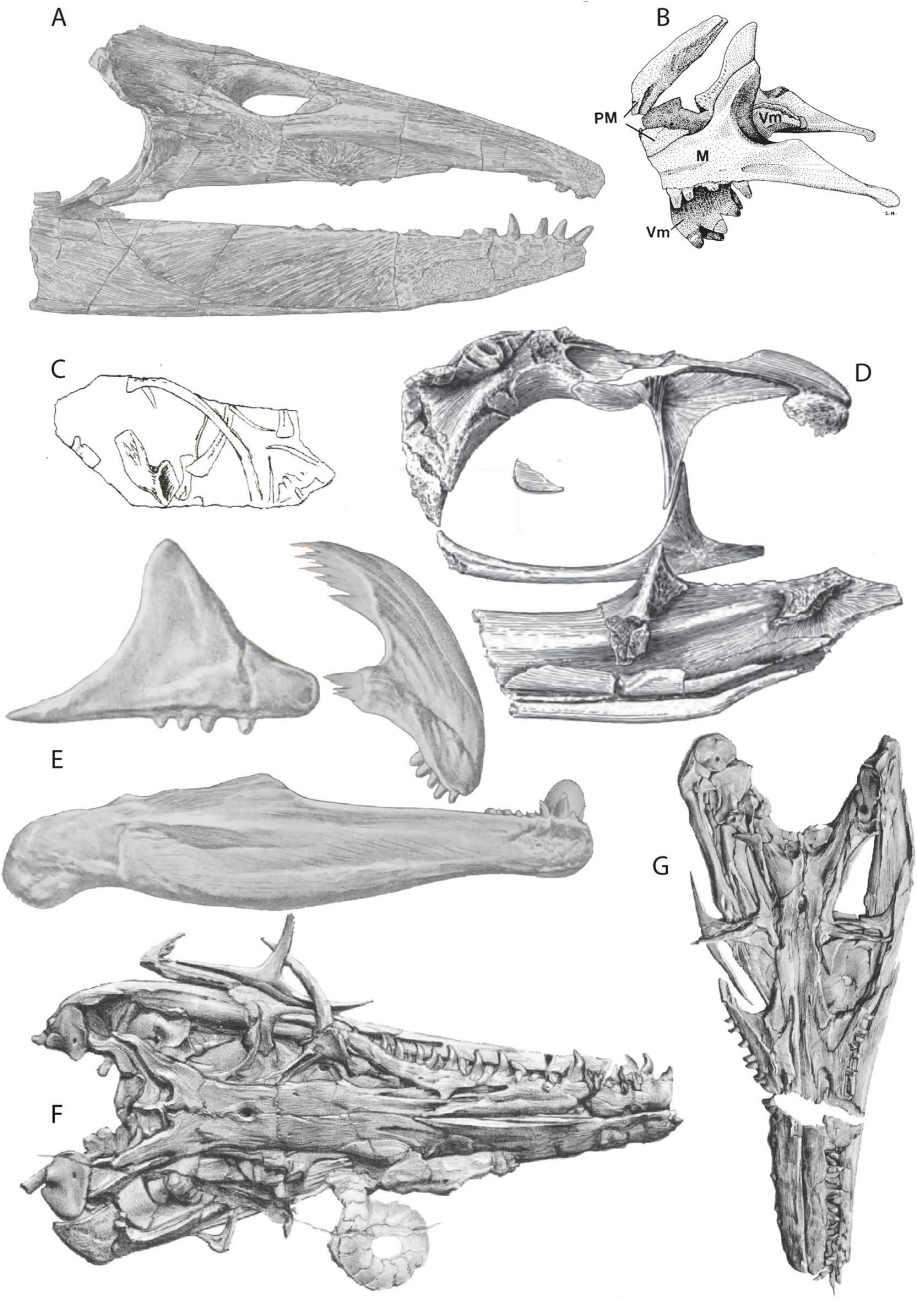
Historic thalattosaur discoveries. **A** *Thalattosaurus alexandrae* (UCMP 9085, holotype) (Merriam, 1905). **B** *Nectosaurus halius* (UCMP 9124, holotype) (Merriam, 1905). **C** *Askeptosaurus italicus* (MSNM V3550, holotype) (Nopcsa, 1925). **D** *Nectosaurus* sp. (UCMP 9120) (Nicholls, 1999). **E** *Hescheleria ruebeli* (PIMUZ T 2469, holotype) (Peyer, 1936b). **F** *Askeptosaurus italicus* (PIMUZ T 4831) (Kuhn, 1952). **G**) *Askeptosaurus italicus* (MSNM V456) (Kuhn, 1952). Drawings are not to scale

Thalattosaur research is marked by periods of paucity alternating with short-term increased interest linked to new discoveries. Initial discoveries of thalattosaur remains date back to the end of the 19th and beginning of the twentieth century and were recovered from the Upper Triassic *Trachyceras* beds of the Hosselkus Limestone in Shasta County, California, USA (Merriam, 1895, 1902, 1904, 1905, 1908). These remains, reflecting the eastern Panthalassic (“Pacific”) Province, are comprised of rare and fragmentary cranial remains and isolated skeletal elements, that represent at least two distinct taxa *Thalattosaurus alexandrae* and *Nectosaurus halius* (Merriam, 1904, 1905, 1908; Nicholls, 1999) (Figs. 1C, 2A, B, D).

Subsequent material was recovered during the 1920s, 1930s and 1950s from the Middle Triassic of Switzerland and Italy, representing the western margin of the Tethys Ocean (Nopcsa, 1925; Peyer, 1936a, 1936b) (Fig. 2C, E). While still representing relatively rare faunal elements in both North American and European Triassic fossil assemblages, the UNESCO World Heritage site of Monte San Giorgio has produced a few more complete and well-preserved specimens. Two out of three genera represented there are solely known from their holotypes. These taxa include *Hescheleria ruebeli* (Peyer, 1936b) and *Clarazia schinzi* (Peyer, 1936a) both discovered in a quarry in Val Porina (Canton Ticino, Switzerland) in 1929 and 1933, respectively, through blasting related to commercial exploitation of the bituminous shales (Peyer, 1936a, 1936b), and *Askeptosaurus italicus* (Nopcsa, 1925). The former two were named after benefactors that supported the excavation activity at Monte San Giorgio of the Zoological Museum of the University of Zürich, Georges Claraz, Professor Dr. H.R. Schinz; and Professor Dr. K. Hescheler who contributed as the director of the Zoological Institute (University of Zurich) to the excavation (Peyer, 1936a; Rieppel, 2019). *Askeptosaurus italicus* was originally described in 1925 based on histological work done by Franz Baron von Nopcsa on a small plate from the Museo Civico di Storia Naturale di Milano. The plate included an ilium, three partial dorsal ribs, a phalanx, half a vertebra and multiple gastralia presumed to be belonging to *Mixosaurus* (Nopcsa, 1925) (Fig. 2C). The distinctive histology, being “*Broomia*-like”, set it apart from other marine reptile groups dismissing a sauropterygian-affinity as suggested by von Heune. The morphology of the pelvic element distinguished it from sauropterygians, ichthyosaurians and archosaurs, perhaps being similar to early archosauromorphs, diapsids such as *Araeoscleidia*, extant squamates and to a lesser degree *Thalattosaurus* (Nopcsa, 1925; Rieppel, 2019). The genus and species name, *Askeptosaurus italicus*, hint at the fact that this new reptile had gone unrecognized by the Italian colleagues in Milan (Nopcsa, 1925). Roughly a dozen partial or relatively complete skeletons were recovered in the thirty years after the erection of the genus (Fig. 2F, G), these together with the skeletons of *Hescheleria ruebeli* and *Clarazia schinzi* provided a first complete glimpse at the detailed skeletal morphology of thalattosaurians. This prompted initial hypotheses on their aquatic capabilities and potentially diverse feeding strategies (Kuhn, 1952; Peyer, 1936a).

A hiatus in prominent publications regarding thalattosaurs in the 1960s and 1970s is indicative of the scarcity of thalattosaurian remains in western deposits of Triassic age (Kuhn-Schnyder, 1960, 1971, 1988). Only a single discovery was made during this time, a well-preserved and articulated claraziid preorbital skull fragment, and associated partial mandible, skull roof and braincase from the lower Carnian Natchez Pass Formation in Jefferson County, Nevada (Storrs, 1991b; Sues & Clark, 2005; H.-D-., Sues Pers. Comm.). Canadian expeditions to the Pink Mountain in northeastern British Columbia during the early 1980s revealed the potential for thalattosaurian material in the Norian Pardonet Formation (Storrs, 1991b). Subsequently a virtually complete skeleton and isolated as well as articulated postcranial material of *Endennasaurus acutirostris* was recovered from the Norian of the Lombardian pre-Alps near Zogno, northern Italy (Müller et al., 2005; Paganoni & Pandolfi, 1989; Renesto, 1984, 2005). Additional works include a redescription of the “claraziids” *Hescheleria* and *Clarazia* by Rieppel (1987). Additional expeditions to British Columbia in the 1980s and 1990s, specifically to the Triassic deposits of the Sulphur Mountain Formation near Wapiti Lake, recovered numerous thalattosaurian slab specimens. These represent at least three genera—*Agkistrognathus campbelli*, *Paralonectes merriami*, and another species of *Thalattosaurus*, *T. borealis*, potentially spanning the (upper) Lower and Middle Triassic (Nicholls & Brinkman, 1990, 1993). Another thalattosauroid, *Wapitisaurus problematicus*, was also recovered during this time but assigned to the Weigeltisauridae instead (Bastiaans et al., 2023; Brinkman, 1988). This relatively productive period was followed by a decade of isolated and fragmentary finds, including isolated anterior caudals similar to *Endennasaurus* from the early Carnian Limestone Formation of the Julian Alps (Dalla Vecchia, 1993); a potential thalattosaurian interclavicle from the Anisian Favret Formation of the Augusta Mountains of Nevada (Sander et al., 1994); various cranial and mandibular remains from the Norian Pardonet Formation of Pink Mountain (Storrs, 1991b), Jewitt Spur and Hudson’s Hope near Williston Lake British Columbia (ROM Pers. Obs.; K., Seymour Pers. Comm.); and a potential thalattosaurian tail fragment from the late Ladinian Spanish Muschelkalk of Tarragona, Spain (Rieppel & Hagdorn, 1998). Shortly thereafter Nicholls (1999) published a revision of the original North American thalattosaur material.

With the discovery of numerous, relatively three-dimensionally preserved specimens from the Middle and Upper Triassic deposits of southwestern China at the onset of the twenty-first century, a potential wealth of information was recovered. The high quantity and diversity of thalattosaur remains recovered from the Upper Triassic Lagerstätten of China, sparked a renaissance in research with the erection of thirteen new taxa in a timespan of roughly twenty years. This renewed interest in thalattosaur in-group relationships, their position within Diapsida, morphological variability, and general functional ecology and biology (e.g. Bastiaans et al., 2023; Benton et al., 2013; Cheng, 2003; Cheng et al., 2007a, 2007b, 2011; Druckenmiller et al., 2020; Jiang et al., 2004; Liu, 1999, 2007, 2013; Liu & Rieppel, 2001; Liu et al., 2013; Maisch, 2014; Metz, 2019; Müller, 2002, 2005; Müller et al., 2005; Rieppel, 2019; Rieppel & Liu, 2006; Rieppel et al., 2000, 2005; Yin et al., 2000; Zhao et al., 2013). In fact, thalattosaurs represent one of the most common and diverse faunal components in these ecosystems starkly contrasting the more sparse and infrequent findings of relatively complete or well-preserved material in Europe and North America (Benton, 2014; Liu et al., 2013).

New thalattosaurian material and possibly new taxa continue to be discovered emphasizing the high potential for more material in historic and other, poorly explored, Triassic marine deposits and the need for further exploration (Storrs, 1991b; Dalla Vecchia, 1993; Nicholls & Brinkman, 1993; Rieppel & Hagdorn, 1998; H.-D-., Sues Pers. Comm.; Müller, 2007; Adams, 2009; Benton et al., 2013; Metz et al., 2015, 2016; Druckenmiller et al., 2016, 2020; Metz, 2019; Čerňanský et al., 2020; Chai et al., 2020a, 2020b; 2021).

**Fig. 3:**
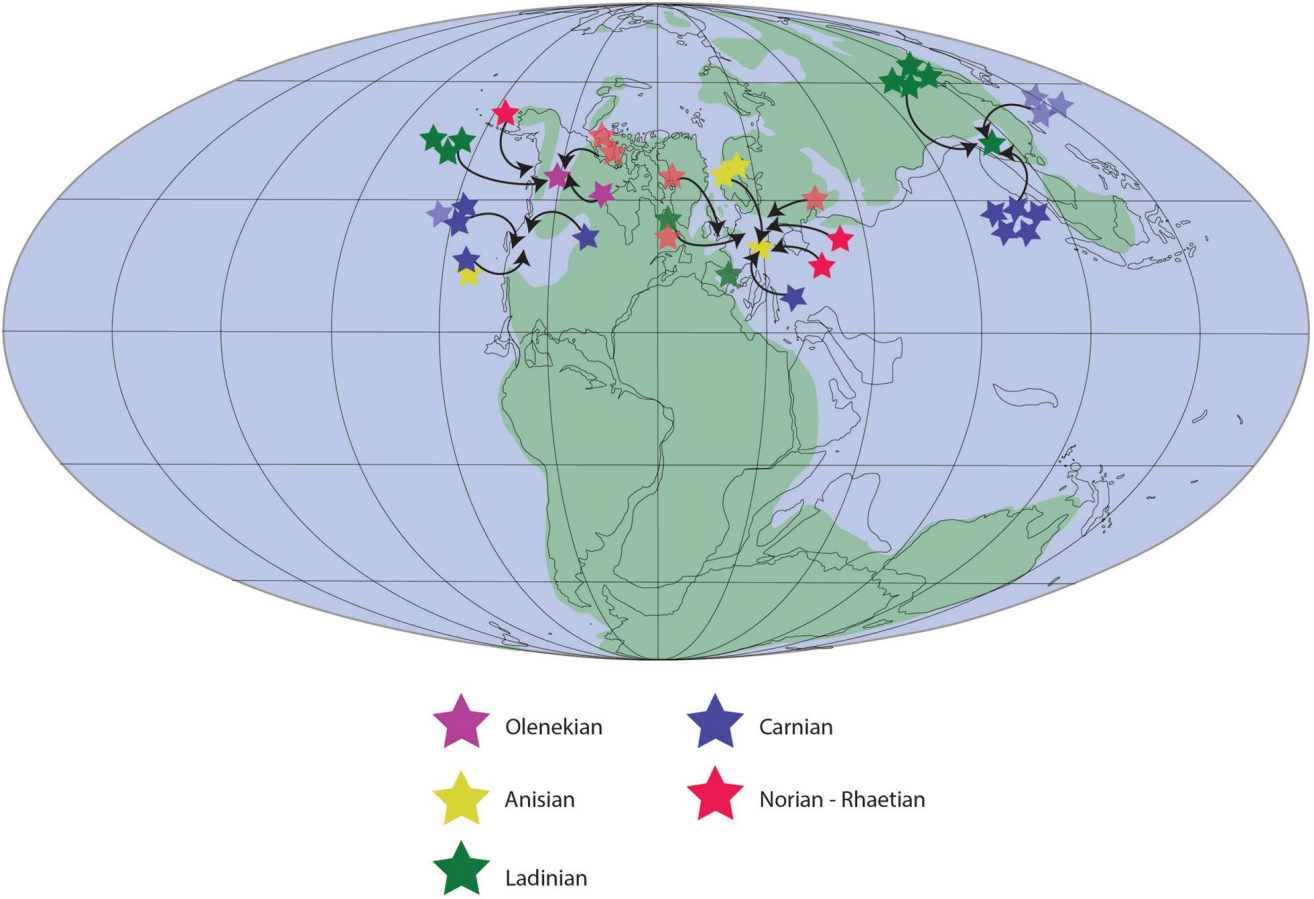
Spatiotemporal spread of thalattosaur species. Solid stars indicate described or recognized species; translucent stars represent postulated material that may be referable to Thalattosauriformes. Map modified from Spiekman and Scheyer (2019), for detailed information see Table 1

Overall, thalattosaur remains have been described from 14 to 17 regions in 6–10 countries, representing 15–18 formations potentially spanning the Olenekian (Lower Triassic) to the Norian-Rhaetian (uppermost Triassic, Fig. 3 and references therein). Their spatiotemporal distribution clearly shows that thalattosaurs are currently most diverse during the Ladinian and Carnian with 5–8 and 6–14 taxa, respectively, compared to 3 taxa in the Anisian, 3–4 taxa in the Norian, and 0–2 taxa in the Rhaetian. Whether this represents a true signal or is related to sampling efforts remains debatable. Given that 2–5 and 3–5 localities represent the Ladinian and Carnian, respectively, and only 2 localities for the late Early Triassic, 3–5 for the Anisian, 3–5 for the Norian and 0–2 for the Rhaetian, very little can be said about changes in diversity (Fig. 3 and references therein). Similarly, thalattosaurs originally seemed to represent rare occurrences in Triassic marine ecosystems, however, the Lagerstätten in China and fragmentary and newly discovered material clearly show that this may not necessarily be true for all localities.

### Evolutionary relationships of Thalattosauriformes

The evolutionary relationships of Thalattosauriformes to other Mesozoic marine reptiles, their position among extant and extinct diapsids and even the monophyletic nature of the clade, have been (and continue to be) the subject of a long history of debate and ambiguity (Kelley, 2012; Kuhn-Schnyder, 1988; Müller, 2005; Rieppel, 2019; Sun et al., 2020). These issues stem from a poor knowledge of their anatomy, their highly derived cranial morphology, and potentially high degree of character reversals and/or convergences with other clades of diapsid reptiles (Rieppel et al., 1987; Kuhn-Schnyder, 1988).

Initially, the Thalattosauria, comprising *Thalattosaurus* and *Nectosaurus*, were considered to be a distinct type of diapsid (“Diaptosauria”) marine reptile, “strongly suggestive of ‘Rhynchocephalia’” (i.e. a wastebasket clade at the time) by Merriam (1904, 1905) (Kuhn-Schnyder, 1988). However, Merriam (1904) did note that thalattosaurs are so different from “typical forms”, and in fact from all other reptilian families, that they “cannot be included in the same ordinal division… and must be given an independent position”, thereby erecting the Thalattosauridae and Thalattosauria clades (Kuhn-Schnyder, 1988; Merriam, 1904). Merriam (1905), Von Huene (1910 in: Kuhn-Schnyder, 1988), Williston (1914 in: Kuhn-Schnyder, 1988), Abel (1919 in: Kuhn-Schnyder, 1988) all also considered thalattosaurs diapsid reptiles, often resembling lepidosaurs, variably squamates and “rhynchocephalians”. However, the discussion on whether or not thalattosaurs had retained two temporal fenestrae was sparked by Franz Nopcsa von Felső-Szilvás (1923 in: Kuhn-Schnyder, 1988) who could not identify a superior temporal opening. Nonetheless, following the erection of *Askeptosaurus italicus* by Nopcsa (1925), he re-examined the relationships of the previously described thalattosaurs and assigned *Askeptosaurus* and likely the other taxa to the “older squamates” (Älteren Squamaten) (Nopcsa, ; Rieppel, 2019). Romer (1933 in: Rieppel, 2019) considered the Thalattosauria to be members of the Eosuchia, a collection of various clades of diapsid reptiles such as younginids and rhynchocephalians (i.e. Sphenodontia + Rhynchosauria), albeit with great hesitation (Romer, 1933 in: Rieppel, 2019).

The description of the thalattosauroids *Clarazia schinzi* (Peyer, 1936a) and *Hescheleria ruebeli* (Peyer, 1936b) came with the erection of a new family, the Claraziidae, which Peyer (1936a, 1936b) postulated had affinities to marine Tocosauria (sensu Fürbringer, 1900), a clade including squamates, rhynchocephalians (including Protorosauria), Ichthyopterygia and “intermediate forms” (Kuhn-Schnyder, 1988; Peyer, 1936a, 1936b; Rieppel, 2019). He suggested that the cranial and dental architecture very likely indicated a common ancestry of *Thalattosaurus* and *Clarazia*, thus including the latter in the Thalattosauria (Peyer, 1936a; Rieppel, 2019). However, little was solved with regard to their presumed diapsid affinity, as the temporal region was either absent (*Hescheleria*) or only ventrally exposed (*Clarazia*) (Peyer, 1936a, 1936b). He suggested that *Clarazia* only possessed an upper temporal opening and that the lower was non-homologous to that of other diapsids, thereby questioning the trait’s taxonomic relevance (Kuhn-Schnyder, 1988; Peyer, 1936a, 1936b). Romer subsequently restructured his Eosuchia to include younginiforms (including prolacertiforms), choristoderes and the thalattosaurs, excluding his Rhynchocephalia and thereby the claraziids (Romer, 1945 In: Rieppel, 2019).

Kuhn (1952) also regarded *Askeptosaurus*, the sole member of the newly erected Askeptosauridae, to be a lepidosaur, specifically, specialized representatives of the early squamates, and presumably related in some way to *Thalattosaurus*, *Clarazia* and *Hescheleria*. He regarded them as having two temporal openings and a streptostylic quadrate but later rescinded both statements (Kuhn-Schnyder, 1988). Romer (1956, 1966 in: Kuhn-Schnyder, 1988) reiterated the inclusion of Thalattosauria, within the Eosuchia alongside clades such as younginiforms, choristoderes, and a separate Prolacertiformes in his latter work (Kuhn, 1952; Romer, 1956, 1966 both in: Kuhn-Schnyder, 1988 and Rieppel, 2019). As a result, thalattosaurs were considered closely related to prolacertiforms and were positioned close to the origin of squamates (Kuhn-Schnyder, 1999; Rieppel, 2019).

Romer, unlike Hoffstetter (1955), doubted the affinity between *Clarazia* and *Hescheleria* and the fidelity of his placement of the Claraziidae into Rhynchocephalia (Sphenodontia + Rhynchosauria) (Romer, 1956, 1966 in: Kuhn-Schnyder, 1988 and Rieppel, 2019). Peyer & Kuhn-Schnyder (1955b in: Rieppel, 2019) inversely included *Askeptosaurus* within the “Triassic squamates” alongside the archosauromorphs *Macrocnemus* and *Tanystropheus*, while *Thalattosaurus* and *Nectosaurus* represented a divergent order related to the Rhynchocephalia (Hoffstetter, 1955 in Rieppel, 2019). Two years later, Romer emphasized the diapsid condition of *Askeptosaurus* and its inclusion within the Eosuchia, while deeming *Clarazia* and *Hescheleria* as indeterminate lepidosaurs, likely members of the Rhynchocephalia (Romer, 1968 in Rieppel, 2019).

With the description of the ventral skull of *Askeptosaurus italicus*, differences in dentition between it and *Thalattosaurus* became more evident (Kuhn-Schnyder, 1971). Kuhn-Schnyder still considered the Thalattosauroidea and the new suborder of Askeptosauroidea as eosuchians with a distinct origin from other representatives of the clades such as squamates and rhynchocephalians (Kuhn-Schnyder, 1971, 1988; Rieppel, 2019). The idea of the Claraziidae being a separate clade from the Thalattosauria persisted for decades. The inclusion of *Clarazia*, and to some extent that of *Hescheleria*, into the Thalattosauria was definite after preparation of the dorsal skull roof of the former, as it clearly shared numerous cranial characteristics with other thalattosaurs, such as the absence of a clear upper temporal fenestra (Rieppel, 1987, 2019).

The introduction of cladistic methods resulted in the restructuring, rearrangement, or complete abandonment of many of the historic paraphyletic or polyphyletic diapsid orders and suborders, such as Tocosauria, Lacertilia, Rhynchocephalia and the Eosuchia (Rieppel, 2019). During the mid- to late 1980s discussions on diapsid taxonomy fueled the debates on thalattosaur affinities within Diapsida (Rieppel, 2019). In fact, Rieppel (1987) illustrates how the strongly divergent morphology of thalattosaurs complicates an assignment to either Lepidosauromorpha or Archosauromorpha and that both are plausible. All three families of Thalattosauria: Thalattosauridae, Askeptosauridae and Claraziidae were considered (early diverging) diapsids (Caroll, 1987 in Kuhn-Schnyder, 1988; Rieppel, 1987). The former two were previously determined to be indeterminate neodiapsids by Benton (1985), while the latter was considered to be? Diapsida incertae sedis. However, despite earlier statements on their diapsid conditions (1952; 1971), Kuhn-Schnyder (1988) considered the possibility that the Thalattosauria do not represent diapsid reptiles (Sauria) and plesiomorphically merely had a lower temporal opening (i.e. no secondarily closed upper temporal fenestra).

Initially described as a lepidosaurian (Renesto, 1984), *Endennasaurus acutirostris* was later reassigned as an edentulous thalattosaur (Renesto, 1991; Rieppel, 2019). Rieppel (1998 in: Müller, 2002) believed thalattosaurs to represent the sister group to sauroptergians within lepidosauromorphs. Both assignments to Lepidosauromorpha (Rieppel, 1998, Carroll & Currie, 1991, both in Müller, 2002) and Archosauromorpha (Evans, 1988; Merck, 1997 in Müller, 2002), the latter of which hinted at close affinities with Sauropterygia and Ichthyopterygia (Merck’s “monophyletic euryapsids”), were proposed during the late 1980s and 1990s. Müller (2002) considered thalattosaurs to be monophyletic and likely positioned with Ichthyopterygia outside of Sauria or otherwise very early diverging within the latter.

Recent phylogenetic analyses, however, indicate a close relationship between thalattosaurs, sauropterygians and saurosphargids. Hypotheses include a sister group relationship between sauropterygians and (thalattosaurs + saurosphargids) (Chen et al., 2014; Li et al., 2011), thalattosaurs and (sauropterygians + saurosphargids) (Li et al., 2014; Wang et al., 2022), Ichthyosauromorpha as sister to (Thalattosauria + Sauropterygia) (Simões et al., 2022), thalattosaurs as a sister group to (Ichthyopterygia + Sauropterygia) (Motani et al., 2015) or to (Ichthyosauromorpha (*Helveticosaurus* + Sauropterygomorpha) (Qiao et al., 2022; Wolniewicz et al., 2023) possibly within a monophyletic Archelosauria together with Archosauromorpha and Testudines (Wolniewicz et al., 2023), or them representing the sister clade to Sauria (Müller, 2004, 2005) (Naish, 2023; Rieppel, 2019).

Neenan et al., (2013) as well as Scheyer et al., (2017), and similar to Bindellini & Dal Sasso (2022), recovered a monophyletic ‘marine superclade’ with Ichthyopterygia as the sister group to Thalattosauriformes, which represents the sister group to a clade of (*Helveticosaurus* (*Eusaurosphargis* (Sauropterygia))) in a polytomy of modern reptile clades (i.e. Testudines, Lepidosauromorpha, Archosauromorpha). This topologically variable marine superclade may or may not represent artificial clustering due to convergent evolutionary adaptations brought upon by similar selective pressures of a marine lifestyle (Chen et al., 2014; Scheyer et al., 2017). Partial or full exclusion of characters deemed problematic, still results in a grouping of various marine clades including Sauropterygia, Ichthyosauromorpha, and Thalattosauriformes, which together may represent the closest sister group to or at least fall outside Sauria (Chen et al., 2014; Cheng et al., 2022; Scheyer et al., 2017; Simões et al., 2018, 2022). Current consensus is that Thalattosauriformes (sensu Nicholls, 1999) represent a monophyletic group of uncertain affinity within Diapsida or Neodiapsida with presumed close affinity to other clades of uncertain position within Diapsida, such as ichthyopterygians and sauropterygians (Benton, 1985; Müller, 2002; Nicholls, 1999).

### Interrelationships within Thalattosauriformes

Nicholls (1999), in the first encompassing study on the interrelationships of thalattosaurs, recovered *Askeptosaurus* and *Endennasaurus* as the earliest branching members of this clade. She defined the Thalattosauria (Merriam, 1905) as “a stem-based group of thalattosauriforms with closer affinity to *Nectosaurus* and *Hescheleria* than to *Endennasaurus* and *Askeptosaurus*” (Sun et al., 2020). The Thalattosauroidea (Nopcsa, 1928) was a “node based group that included the last common ancestor of *Nectosaurus* and *Agkistrognathus* and all their descendants” (Nicholls, 1999). She excluded *Nectosaurus* from the Thalattosauridae (Merriam, 1904), defined as “stem based group” including all thalattosaurians “more closely related to *Clarazia* and *Thalattosaurus* than *Nectosaurus*.”. The Thalattosaurida (Nicholls, 1999) encompasses “the last common ancestor of *Thalattosaurus*, *Paralonectes* and *Agkistrognathus* and all their descendants” (Nicholls, 1999).

Rieppel et al., (2000) found two discrete clades: a clade including *Askeptosaurus* and the Chinese *Anshunsaurus* (Askeptosauroidea) and an unresolved clade containing *Clarazia*, *Hescheleria* and *Thalattosaurus* (Thalattosauroidea), a split that had been recovered by previous analyses by Kuhn-Schnyder (1971), Rieppel (1987) and Nicholls (1999). Similar results were obtained by analyses of Müller et al., (2005) with *Endennasaurus* as the sister clade to the Askeptosauridae, consisting of *Askeptosaurus* and *Anshunsaurus*. Liu & Rieppel. (2001) and Jiang et al., (2004) however did not find a monophyletic Askeptosauridae. The analyses of Müller (2002, 2005) represent the first larger scale endeavors to unravel ingroup relationships of thalattosaurs, which recovered a monophyletic Askeptosauroidea, a monophyletic *Xinpusaurus* and *Nectosaurus* clade, and a monophyletic *Clarazia* and *Hescheleria* clade (Müller, 2005).

The taxonomy of the recently described Chinese taxa needs detailed revision as the validity of several taxa is contentious and thus the diversity and disparity of Late Triassic thalattosaurs is incompletely understood (Rieppel, 2019; Maisch, 2024). Some species may reflect ontogenetic morphs of the genotypical species (e.g. *X. bamaolinensis* and *X. kohi*, Liu, 2013; Maisch, 2014, 2024; Li et al., 2016; or *Anshunsaurus huangnihensis*, Cheng et al., 2007a, 2011; Zhao et al., 2008; Benton et al., 2013; Chai et al., 2020b; Maisch, 2024), while other putative thalattosaurs such as *Neosinasaurus* and *Wayaosaurus* may be closely related or synonymous to the previously described taxon *Miodentosaurus* (Chai & Jiang, 2021; Chai et al., 2023; Wu et al., 2009). The re-identification of the presumed maxilla in *Concavispina* (ZMNH M8804) as a vomer, increases the resemblance between it and other taxa, prompting questions about a possible congeneric relationship with *Xinpusaurus* (personal observations based on: Liu et al., 2013; Rieppel, 2019; Zhao et al., 2013).

Furthermore, the exact position of taxa within the Askeptosauroidea remains unresolved, with for instance *Askeptosaurus* representing a sister taxon of *Miodentosaurus* (Wu et al., 2009), *Endennasaurus* (Cheng et al., 2011) or *Anshunsaurus* (Li et al., 2016; Liu & Rieppel, 2005; Liu et al., 2013; Müller, 2002, 2005, 2007), resulting in either a monophyletic or paraphyletic *Anshunsaurus* (Druckenmiller et al., 2020; Li et al., 2016), or a polytomy of all askeptosauroids or all *Anshunsaurus* species (Cheng et al., 2011; Druckenmiller et al., 2020; Liu et al., 2013; Metz, 2019). The position of *Miodentosaurus* also is highly variable, showing similar spread as *Askeptosaurus* within the Askeptosauroidae, including a sister taxon relationship with *A. huangguoshuensis* in a paraphyletic *Anshunsaurus* clade (analysis of Druckenmiller et al., 2020 with the exclusion of *A. wushaensis*). The relationships in Thalattosauroidea are equally unstable, with weak branch support and often poorly resolved relationships (Liu & Rieppel, 2001). Re-occurring patterns in most recent phylogenetic analyses are the grouping of the eastern Tethyan thalattosauroids into a single monophyletic clade (e.g. Druckenmiller et al., 2020; Li et al., 2016; Liu & Rieppel, 2005; Wu et al., 2009) and perhaps the early diverging position of *Agkistrognathus* and *Paralonectes* (Liu & Rieppel, 2005; Li et al., 2013, 2016).

Current views on ingroup relationships of Thalattosauriformes support the traditional subdivision into two monophyletic (super)families: the Askeptosauroidea and the Thalattosauroidea (Müller, 2002, 2005, 2007; Liu & Rieppel, 2005; Wu et al., 2009; Cheng et al., 2011; Liu et al., 2013; Li et al., 2016; Druckenmiller et al., 2020; Jiang et al., 2023; Maisch, 2024). Thalattosaur interrelationships beyond a monophyletic Askeptosauroidea and Thalattosauroidea remain troublesome and unstable, likely reflecting the highly derived morphology of many taxa, and perhaps influences of homoplasy and reversals. More detailed morphological information is needed on many of the historic taxa as aspects of their cranial anatomy are still poorly understood.

### Thalattosaurs in time and space

The unresolved phylogenetic relationships of thalattosaurs within Diapsida and the unresolved interrelationships within Thalattosauriformes itself, combined with their spotty and biased fossil record, make it near impossible to reconstruct their paleobiogeographic evolution at this stage. Only general patterns of dispersal and global spread can reliably be outlined and discussed (Rieppel et al., 2000; Cecca, 2002 in: Bardet et al., 2014).

#### Early Triassic

During the earliest stages of the Early Triassic periodic extreme heating made life on the continent in much of the low latitudes unsustainable, while the oceans preserve a narrow habitable zone between hot surface waters and anoxic bottom waters (Benton, 2016; Benton et al., 2013). At the time equatorial ecosystems consist of predominantly small invertebrate faunas (Benton, 2016; Benton et al., 2013). The middle-late Smithian and Spathian saw less extreme climatic and oceanic environmental conditions allowing marine reptiles, especially ichthyosauromorphs followed by sauropterygians, to rapidly radiate and attain a wide distribution and taxic diversity (Bardet et al., 2014; Benton et al., 2013; Galasso et al., 2022; Hu et al., 2024; Jiang et al., 2023; Kear et al., 2023; Sander, 2000; Scheyer et al., 2014; Storrs, 1991a; Wang et al., 2022). Almost thirty species of marine reptiles have been recovered globally within 2 million years into the Early Triassic (Du et al., 2023; Jiang et al., 2023). Many Early Triassic taxa were shallow water species along the Tethyan or Panthalassan shorelines (Bardet et al., 2014; Benton et al., 2013; Du et al., 2023; Jiang et al., 2023; Neenan et al., 2013; Sander, 2000). These recovering early marine ecosystems were predominantly constituted of small to medium-sized marine reptiles with rare large-bodied taxa (Du et al., 2023; Jiang et al., 2023). Despite the presence of superficially, and presumed functionally, somewhat similar reptiles in Lower Triassic localities worldwide, no definite thalattosaurs are known from these or contemporaneous deposits with the possible exception of British Columbia (Bastiaans et al., 2023; Benton et al., 2013; Sander, 2000).

The earliest representatives of the Thalattosauriformes might come from the Lower Triassic (Olenekian) of North America (Fig. 6A). Four genera, namely *Thalattosaurus borealis*, *Paralonectes merriami*, *Agkistrognathus campbelli* and *Wapitisaurus problematicus*, are documented from the Lower-Middle Triassic Sulphur Mountain and Meosin Mountain formations of British Columbia, western Canada (Bastiaans et al., 2023; Nicholls & Brinkman, 1993; pers. obs.). While numerous specimens were discovered as float on scree slopes, making it challenging to determine their precise age, some specimens of *Paralonectes*, possibly *Agkistrognathus*, and indeterminate thalattosaur remains have been originally postulated to be from the Olenekian and more specifically the Smithian based on lithology (Orchard & Zonneveld, 2009; Wendruff & Wilson, 2013). Others such as the holotype of *Agkistrognathus campbelli*, the holotype of *Paralonectes merriami*, the referred specimens of *P. merriami*, Thalattosauridae indet (cf. *Thalattosaurus*), and various remains of indeterminate thalattosaurians were recovered from the Olenekian to Middle Triassic 'cirque D' locality of Ganoid ridge (Nicholls & Brinkman, 1993). *W. problematicus* (TMP 86.153.14), although recovered as scree material, can reasonably but tentatively be assigned to the Olenekian siltstone layers of the Vega-Phroso Member of the Sulphur Mountain Formation at Ganoid Ridge, Wapiti Lake area based on lack of associated invertebrate content and its lithology (Bastiaans et al., 2023). Nonetheless, additional geochemical, sedimentological and microfossil analyses need to be undertaken in order to unequivocally identify the source members of the various ex situ and tentative Lower Triassic thalattosaur remains from the Sulphur Mountain and Meosin Mountain formations (Bastiaans et al., 2023).

The poor preservation of these specimens, being disarticulated, heavily eroded and flattened, make detailed descriptive work difficult and their exact identification uncertain. Nonetheless, some preliminary observations can be made. Many of the dentigerous elements of these thalattosaurs already display a high degree of heterodonty with small and large conical symphyseal teeth and more durophagous and progressively larger dentition that is increasingly ventromedially offset along the dental margin. Associated cranial and body length estimates range between 10–15 cm and 1–2 m, respectively (based on Nicholls, 1999). The presence of *W. problematicus* and possibly coeval *Paralonectes* sp. and various indeterminate thalattosaur remains from Meosin Mountain (RTMP collections), may hint at a previous underestimation of early thalattosauroid diversity and disparity. *Wapitisaurus* seems surprisingly morphologically derived, sharing several characteristics with Middle-Late Triassic thalattosauroids (Bastiaans et al., 2023). In addition, the posterior position of the external nares, and the potentially high abundance of thalattosauroids in Lower Triassic (shallow) deposits of British Columbia may indicate an earlier marine invasion than previously assumed (Bastiaans et al., 2023). Hypotheses such as an early burst pattern that may have paralleled those seen in early ichthyopterygians (Kear et al., 2023; Moon & Stubbs, 2020) and sauropterygians (Wang et al., 2022) with widespread opportunistic trophic niche diversification in the shallow marine realm relatively rapidly after the EPME, will require additional data and further testing. During much of the Early Triassic thalattosaurs hypothetically may have represented relatively uncommon (but certainly not rare) occurrences in (a few geographically restricted) ecosystems alongside numerous small mixosaurs and indeterminate ichthyosaurs (Callaway & Brinkman, 1989; Schaeffer et al., 1976). Alternatively, as similarly proposed for ichthyosauromorphs and sauropterygians, the fossil record may obscure much of their early diversification history and their diversity and degree of aquatic specialization accumulated over an unknown interval of time prior to the Olenekian. However, more data is needed to unequivocally determine their abundance and biogeographic distribution.

#### Middle Triassic

During the Middle Triassic, ecosystems slowly stabilized and complex trophic networks were established with some of the highest diversity of marine reptiles of the entire Mesozoic (Bardet, 1994; Kelley et al., 2014; Neenan et al., 2015; Benton, 2016; Widmann et al., 2020; Jiang et al., 2023). By the (late) Middle Triassic, a global transgression allowed thalattosaurs to radiate and achieve a near-cosmopolitan distribution (Fig. 6B, C), inhabiting the low-latitude tropical shallow marine environments along the eastern (southern China) and western (central Europe) Tethyan and eastern Panthalassic (western North America) margins (Bardet et al., 2014; Druckenmiller et al., 2020; Rieppel et al., 2000; Sun et al., 2020). How thalattosauroids crossed from the eastern to the western margin of the Panthalassa remains unclear (Sun et al., 2020). The absence of an Atlantic passage between mid to low latitudes only allows for two remaining hypotheses: (I) a coastal or coastal-pelagic migration along the northern margin of Laurasia, through the colder, high latitude waters (“Boreal route” of e.g. Hallam, 1994 in: Bardet et al., 2014); or (II) a direct pelagic dispersal between the east and west coast of Panthalassa via equatorial refuges has occurred but remains of such widespread taxa are still to be recovered (Bardet et al., 2014; Müller, 2002; Rieppel et al., 2000; Sun et al., 2020). Dispersal through the high latitude environments seems unlikely due to physiological (see Motani, 2009) and climatic constraints (Rieppel, 2000; Müller, 2002). Thalattosauroids may not have been able to sustain adequate body temperatures in these colder waters in contrast to pelagic Triassic ichthyosaurs (Müller, 2000; Sander, 2000). The climatic conditions in this area during the Early Triassic, however, may have been much warmer than previously assumed, allowing for the early dispersal of ichthyosaurs, sauropterygians and thalattosaurs between the Panthalassic and Tethyan Provinces (Brayard et al., 2007 in Bardet et al., 2014).

##### Anisian

By the Middle Triassic many fossil assemblages, such as the Sulphur Mountain Formation (British Columbia), Upper Saurian Level (Svalbard), Panxian (China) and the Favret Formation (Nevada) show an explosive diversification of, especially durophagous, marine reptiles (Benson et al., 2010; Schmitz et al., 2005). During this time marine transgressions eased faunal interchanges between the eastern and western Tethyan realms (Jiang et al., 2023; Liu et al., 2013; Neenan et al., 2013, 2015; Rieppel, 2019; Sun et al., 2020). Faunal interchanges between the eastern Tethyan ecosystems and the eastern Panthalassic faunas remained quite an undertaking with only taxa adequately adapted to pelagic or coastal-pelagic migrations, such as ichthyosaurs, plesiosaurs, and pistosauroids seemingly being able to easily cross (Neenan et al., 2013).

Thalattosaurs, as opposed to sauropterygomorphs and protorosaurs, do not show a wide peritethyan distribution during the Middle Triassic and are mainly restricted to the Anisian beds of the Besano Formation and Ladinian deposits in southwestern China (Mazin, 2001; Rieppel, 2001; Müller, 2002, 2005; Motani, 2009; Neenan et al., 2013, 2015; Bardet et al., 2014; Neenan, 2014; Renesto & Dalla Vecchia, 2017; Rieppel, 2019; Spiekman & Scheyer, 2019; Druckenmiller, 2020; Klein et al., 2022). To date, no Anisian or Early Triassic remains of thalattosaurs have been recovered from the eastern Tethyan province and robust phylogenetic analyses are necessary to test hypotheses on the specific paleobiogeographic patterns of the clade.

During the late Anisian the western Tethyan basins were inhabited by the 2–3 m long *Askeptosaurus italicus*, and the smaller (~ 1 to 1,5 m long) thalattosauroids *Clarazia schinzi* and *Hescheleria ruebeli* (Fig. 6) (Bardet et al., 2014; Kuhn, 1952; Kuhn-Schnyder, 1960, 1971, 1988; Müller, 2005; Peyer, 1936a, 1936b; Rieppel, 1987, 2019). In the very latest Anisian the fauna slightly changes, *Mixosaurus* and thalattosaurs disappear and pachypleurosauroids and nothosaurids diversify (Peyer, 1936a, 1936b; Müller, 2002; Rieppel, 2019; Spiekman & Scheyer, 2019; Bindellini & Sasso, 2022). At least two genera are present in the eastern Panthalassic province during this time, namely *Agkistrognathus* (TMP 1995.115.1) and *Thalattosaurus borealis* (holotype, TMP 89.126.1). Additional remains from Cirque T (upper Anisian-lower Ladinian) of Wapiti Lake in British Columbia indicate the potential presence of *Paralonectes* (or *T. perrini* according to Nicholls, 1999) (TMP 1989.126.2) and other indeterminate thalattosaurs (e.g. TMP 1991.123.2) alongside various ichthyosaurs (Callaway & Brinkman, 1989; Nicholls & Brinkman, 1993, p. 264; Sander & Mazin, 1993; McGowan, 1997). A potential small thalattosaurian interclavicle (FMNH PR 1803) from the Fossil Hill Member of the Favret Formation in the Augusta Mountains may indicate their presence also in Nevada (USA) during the Anisian (Sander et al., 1994).

##### Ladinian

Despite the lack of unequivocal Ladinian thalattosaurian remains in Europe, their presence may be indicated by putative remains assigned to “*Blezingeria*" from the Germanic basin and a tail fragment from the Spanish Muschelkalk (Fraas, 1896; Rieppel & Hagdorn, 1998; Schoch & Wild, 1999; Schoch et al., 2015). *Blezingeria ichthyospondylus* (Fraas, 1896) from the Muschelkalk/Lettenkeuper likely represents a wastebin taxon comprised of isolated vertebral, pelvic, rib and limb remains of various marine reptiles. Re-examination of multiple specimens by Müller (2002: 122–123) could neither ascertain nor refute the possibility of some of these remains representing thalattosaurs. The 45-cm-long tail fragment from the upper Ladinian Alcover unit of the Spanish Muschelkalk in Tarragona, Spain was postulated to belong to a thalattosaur on the basis of its “elongate and slender neural spines and haemal arches" and close resemblance to caudal remains from the Carnian of Italy that was previously assigned to thalattosaurs (Bardet et al., 2014; Dalla Vecchia, 1993; Rieppel & Hagdorn, 1998). If these putative thalattosaur remains are corroborated by the discovery of additional remains, that may indicate that thalattosaurs diversified in the southern Alpine region and subsequently spread to the western and Peritethys (Rieppel & Hagdorn, 1998). During this time many of the alpine Triassic ecosystems show a highly reduced vertebrate diversity which may partly be correlated to hypersaline conditions (Müller, 2002).

The earliest representatives of thalattosaurs in the eastern Tethys come from the upper Ladinian Zhuganpo Formation (previously Zhuganpo Member of the Falang Formation) near Xingyi City, Guizhou Province (Benton et al., 2013; Jiang et al., 2023; Lu et al., 2018). The Ladinian in South China is marked by a distinct turnover from predominantly ‘Tethyan’ coastal to more pelagic taxa with close affinity to taxa of the Panthalassic ecosystems (Benton et al., 2013; Jiang et al., 2023; Lu et al., 2018; Rieppel, 2019).

A recent discovery of an isolated antorbital cranial fragment of a thalattosauroid from the lower assemblage of Xingyi shows a strongly ventrally deflected premaxillary rostrum (XNGM-WS-22-R5, Chai et al., 2020a, 2020b). Its cranial morphology resembles that of, and shares potentially close affinity with, *Hescheleria ruebeli* from the western Tethys (Chai et al., 2020a, 2020b; Jiang et al., 2023). Its similar paleoenvironment and presumed ecological resemblance and affinity with the claraziids of Monte San Giorgio and the relationship between *Anshunsaurus* and *Askeptosaurus*, again illustrate the close faunal links between the eastern and western Tethyan provinces during the (late) Middle Triassic (Chai et al., 2020a, 2020b; Cheng et al., 2007a, 2011; Jiang et al., 2023; Müller, 2005, 2007; Rieppel et al., 2000, 2006).

Finds from the upper Ladinian deposits of the fourth member of the Gejiu Formation (equivalent to the lower assemblage of the Falang Formation of Guizhou Province) around Niubudai Village, Banqiao, Luoping County in Yunnan Province, include a partial caudal vertebral series and articulated right hindlimb of a 1.5–2 m long askeptosauroid thalattosaur (cf. *Askeptosaurus*) (Benton et al., 2013; Sun et al., 2005). This contrasts the absence of askeptosauroids in the lower assemblage of Xingyi, however, overall the faunal composition of both localities are indistinguishable (Benton et al., 2013).

Thalattosaurs show relatively modest taxic diversity and low morphological disparity in the fauna of the upper assemblage of Xingyi, with at least two genera and potentially four species present, all of which with relatively elongate and straight rostra: three species of the askeptosauroid *Anshunsaurus* (*A. wushaensis* (Rieppel et al., 2006); *A. huangnihensis*, (Cheng et al., 2007a); *Anshunsaurus* cf. *A. huangguoshuensis*, (Chai et al., 2020b)) and a single species of *Xinpusaurus*, *X. xingyiensis* (Li et al., 2016) (Benton et al., 2013; Rieppel, 2019). All are roughly 2–3 m in length (Benton et al., 2013; Chai et al., 2020b; Cheng et al., 2007a, 2007b; Rieppel, 2019; Rieppel et al., 2006).

Towards the end of the Middle Triassic volcanism, a shift to a warm-humid climate and major changes in global sea level and oceanic chemistry (e.g. possible acidification) restructured global marine ecosystems (Bardet, 1994; Benton et al., 2013; Lu et al., 2018; Zhang et al., 2021). A tectonically-driven change into largely pelagic-dominated assemblages in the eastern Tethyan ecosystems is well-illustrated by the Guanling biota, which includes very large ichthyosaurs and thalattosaurs and a lack of benthic and endobenthic taxa (Benton et al., 2013; Lu et al., 2018; Zhang et al., 2021).

##### Carnian

The Carnian marks a time of substantially decreased terrestrial and marine diversity and significant biological turnover amidst major climatic upheavals, changes in the hydrological cycling and perhaps extensive volcanism (Bardet, 1994; Corso et al., 2020). Over one-third of marine genera and > 50% of marine reptile families were lost during this time, followed by emergences and radiations of new clades (Bardet, 1994; Corso et al., 2020). Throughout the Carnian period, thalattosaur diversity seems to experience a significant peak worldwide. This may partially or largely be influenced by lagerstätten effects, despite the high degree of geological sampling for the Carnian to Norian (Benson et al., 2010). Within various ecosystems in China and North America, a total of 9–12 confirmed thalattosaur species, with an additional 2 species currently under study, have been identified. Interestingly, ichthyosaurs may have experienced a relative decrease in diversity (Renesto & Dalla Vecchia, 2017). Whether this decrease is genuine, and due to an abiotic cause or perhaps due to competition for niches with the increasingly disparate and diverse thalattosaurs is currently unclear. The high diversity and disparity of Carnian thalattosaurs may inversely also be a consequence of the relative decline in ichthyosaur diversity, if again it reflects a genuine pattern, or perhaps of changes in feeding ecology in the latter group. The last non-plesiosaurian eosauropterygians likely go extinct during the early or middle Carnian which may also have allowed thalattosaurs to greatly diversify and attain a greater paleoecological diversity (Bardet et al., 2014; Renesto & Dalla Vecchia, 2017).

At least four to six genera and six to nine species of thalattosaur have been recovered from the Carnian fossiliferous beds of the Xiaowa Formation (previously Wayao Member of the Falang Formation) in Guanling county, Guizhou Province, southwestern China: *Xinpusaurus suni*, *X. kohi*, *X. bamaolinensis*, *Concavispina biseridens*, *Miodentosaurus brevis*, *Neosinasaurus hoangi*, *Wayaosaurus bellus*, *W. geei* and *Anshunsaurus huangguoshuensis*, the latter of which may have been present in the late Ladinian already (see Chai et al., 2020b) (Liu, 1999; Rieppel et al., 2000; Yin et al., 2000; Liu et al., 2001; Liu & Rieppel, 2001; Luo & Yu, 2002; Cheng, 2003; Jiang et al., 2004; Liu & Rieppel, 2005; Rieppel & Liu, 2006; Cheng et al., 2007a, 2007b; Wu et al., 2009; Zhao et al., 2010, 2013; Benton et al., 2013; Liu et al., 2013; Maisch, 2014; Li et al., 2016; Rieppel, 2019). The Guanling biota represents a remarkably and unparalleled varied assemblage of contemporaneous thalattosaur species, surpassing any known assemblage from around the globe (Rieppel, 2019). Numerous specimens of the genera *Xinpusaurus* and *Anshunsaurus* have been recovered, making them some of the most abundant faunal components in these Carnian ecosystems (Benton et al., 2013; Liu et al., 2013). The poorly preserved and partially prepared remains of *Neosinasaurus* and *Wayaosaurus* may prove to be closely related or even synonymous to known askeptosauroid thalattosaurs (e.g. *M. brevis*), thereby possibly further increasing/decreasing the thalattosaur diversity by up to two genera and three species (Chai & Jiang, 2021; Chai et al., 2023; Wu et al., 2009; Yin et al., 2000). The faunal similarities between the Ladinian ecosystems from the Upper Assemblage at Xingyi and the Carnian Guanling biota are much greater than previously acknowledged with the genera *Xinpusaurus* and *Anshunsaurus* displaying a longevity of perhaps several millions of years (Benton et al., 2013; Li et al., 2016).

More than 75% of the vertebrate fauna at Guanling is comprised of medium- to large-bodied marine reptiles, which is more than three times as much as during the Spathian (Benton et al., 2013). Taxa are also significantly larger compared to the Lower Triassic Chaohu Fauna, possibly indicating a fully recovered ecosystem (Benton et al., 2013). However, while taxic diversity and disparity are very high among marine reptiles during this time, very little is known about the functional diversity and functional uniqueness, richness and specialization within these ecosystems, in particular with regards to thalattosaurs. Towards the end of the Carnian thalattosaurs such as *Concavispina, Miodentosaurus* and *Anshunsaurus huangguoshuensis* attained body sizes in excess of 3 m and may have occupied apex predatory niches (Cheng et al., 2007a, 2007b; Liu, 1999; Liu et al., 2013; Rieppel, 2019; Rieppel et al., 2000; Wu et al., 2009; Zhao et al., 2013).

An articulated caudal series from the lower Carnian “scisti ittiolitici di Raibl” may indicate the presence of small-sized *Endennasaurus*-like thalattosaurs in the Alpine Triassic during this time (Dalla Vecchia, 1993). This may indicate the persistence of askeptosauroids in the western Tethys after the Anisian. However, the post-Anisian fossil record for thalattosaurs of this region is too incomplete and the assignment of this specimen to Thalattosauriformes is too uncertain to make unequivocal interpretations.

In the eastern Pacific, numerous cranial and postcranial remains of thalattosauroids have been collected from the lower Carnian inferior member of the Natchez Pass Formation of Humboldt County, northwestern Nevada (Sues & Clark, 2005). Although currently under study, at least one new genus of “claraziid” has already been identified based on an articulated cranium and referred basicranial remains (Sues, H-.D. pers. Communication; Storrs, 1991b; Sues & Clark, 2005). This thalattosauroid strongly resembles *Hescheleria ruebeli* and XNGM-WS-22-R5 in that its premaxillary rostrum is strongly ventrally deflected (beyond 90° relative to the horizontal plane) (Chai et al., 2020a, 2020b; Peyer, 1936a, 1936b; Rieppel, 1987; Sues & Clark, 2005). However, it differs from these taxa in that its premaxillary dentition consists of pseudodont osseous outgrowths as has been suggested for *Thalattosaurus alexandrae* (Nicholls, 1999; Sues & Clark, 2005). Detailed anatomical and phylogenetic work needs to establish its position and interrelationships in Thalattosauriformes, but its presence may hint at faunal connectivity and faunal interchanges between all three Provinces during the Middle and early Late Triassic (Rieppel, 2019; Sues & Clark, 2005).

Three to four additional taxa are identified from the upper Carnian deposits of California and Oregon: *Thalattosaurus alexandrae*, *Nectosaurus halius*, an undescribed taxon referred to here as the Brisbois Member taxon and if considered a valid taxon, *T. perrini* (Merriam, 1905; Metz, 2019; Metz et al., 2016; Nicholls, 1999).

The Hosselkus Limestone of Shasta County, California (USA), is an extremely rich faunal deposit with small (< 2 m, e.g. *Torectocnemus*), medium- and large-sized (> 7 m shastasaurids) ichthyosaurs, and various thalattosauroids (Merriam, 1902, 1904, 1905, 1907, 1908; Nicholls, 1999). *Thalattosaurus alexandrae* is a medium-sized early diverging thalattosauroid measuring between 2 and 3 m in length (Druckenmiller et al., 2020; Merriam, 1905; Nicholls, 1999). Another species of *Thalattosaurus* may be present in the Shasta County fauna, *T. perrini*, however, this poorly described taxon had been lost for over a century and has only recently been recovered (Nicholls, 1999; Pers. Obs.). This genus seemingly is particularly long-lived, potentially spanning the Olenekian or at least the Ladinian to the Carnian, (Müller, 2002; Nicholls & Brinkman, 1993). The second genus of thalattosaur in these deposits is the smaller (~ 1 m) *Nectosaurus*, however, much larger referred specimens exist (e.g. *Nectosaurus* sp., UCMP 9120) (Merriam, 1905, 1908; Naish, 2023; Nicholls, 1999). Although only represented by fragmentary cranial and postcranial material, this taxon likely possessed a strongly ventrally deflected rostrum and heterodont dentition (Nicholls, 1999). Given its position as the sister taxon to a clade including all Tethyan thalattosauroids in the most recent phylogenetic analyses (Druckenmiller et al., 2020; Jiang et al., 2023), this taxon may be very important in determining the paleobiogeographic history of these faunas and the development of ventrally deflected rostra.

Multiple (≥ 5) individuals of different ontogenetic stages of a large early diverging thalattosauroid genus have been recovered from a calcareous conglomerate nodule of the Brisbois Member of the Vester Formation of central Oregon (Metz et al., ; Metz, 2019). This shallow nearshore setting in the forearc of the Izee Terrane additionally produced fossils of hybodontid sharks, colobodontid fish, indeterminate marine tetrapod (perhaps another thalattosaur), an archosaur and an ichthyosaur (Metz et al., ; Metz, 2019). This taxon shows a moderately ventrally deflected rostrum slightly more pronounced than that observed in *Thalattosaurus* and sharp, relatively homodont conical teeth (Metz, 2019).

##### Norian and Rhaetian

Towards the end of the Triassic several marine reptiles seem to show a prominent decrease in disparity and diversity or perish altogether, which defined the macroevolutionary history for surviving groups for the remainder of their existence (Thorne et al., 2011; Kelley et al., 2014; Renesto & Dalla Vecchia, 2017; Moon & Stubbs, 2020). Substantial transgression between early and late Carnian was followed by a major regression, which led to the lowest quantity of flooded continental shelf during the Mesozoic, from the late Carnian onwards. This resulted in a progressive loss of shallow marine habitats, and consequently many durophagous taxa, towards the end of the Triassic (Benson & Butler, 2011; Benton et al., 2013; Kelley et al., 2014; Stubbs & Benton, 2016; Renesto & Dalla Vecchia, 2017; Druckenmiller et al., 2020). A short marine transgression during the middle to late Norian submerged large parts of the Alpine realm, which has led some authors to suggest that this area could have served as refugium for the last northwestern Tethyan coastal and coastal pelagic marine reptiles, such as placodonts and thalattosaurs (Müller, 2002; Kelley et al., 2014; Renesto & Dalla Vecchia, 2017). The rifting of the North Atlantic Ocean and outgassing of the Central Atlantic Magmatic Province resulted in a distinct climatic shift towards arid and hot conditions and several widespread pulses of extinction (Benton, 2005, 2014, 2016; Cawthorne et al., 2024). Thalattosaurs, although still present after the Carnian, seem to show very little cranial disparity, with the sole presence of taxa with straight, elongated, ichthyosaur-like crania. If representing a genuine signal, this pattern may reflect relatively narrow ecological diversity for remaining thalattosaur taxa (Druckenmiller et al., 2020; Müller, 2007; Müller et al., 2005; Sander, 2000). However, relative taxic diversity may be underestimated due to the relatively poor Carnian, and especially the Norian-Rhaetian fossil records for marine reptiles (Benson & Butler, 2011; Benson et al., 2010; Moon & Stubbs, 2020; Thorne et al., 2011), as may also be illustrated by the widespread occurrence of thalattosaurs in Alaska, Canada, Italy and Austria during that time (Druckenmiller et al., 2020; Motani, 2009; Müller, 2007; Müller et al., 2005; Paganoni & Pandolfi, 1989; Renesto, 1984, 2005; Storrs, 1991b).

The deep-water ecosystems of the Pardonet Formation of British Columbia along Williston Lake and in the Pink Mountain preserves a rich marine reptile fauna, including numerous ichthyosaurs and rare thalattosaurians (McGowan, 1997; Nicholls & Manabe, 2004; Wignall et al., 2007). Besides partial cranial remains and articulated skeletal material from Jewitt Spur at Williston Lake (Pers. Obs. RTMP), isolated remains of limbs, caudal vertebrae and ribs have been recovered from the Pink Mountain in northeastern British Columbia (Storrs, 1991b). Thalattosaurs, seem to have remained relatively restricted in length with partial mandible lengths of < 10 cm, possibly indicative of body sizes between 1 and 2 m. The mandibular dentition of the better-preserved ROM 46211 seems to resemble that of *Gunakadeit joseeae* from the middle Norian of the Keku Islands, southeastern Alaska, in both size and morphology, being small, relatively homodont, with slender, conical and recurved teeth (Druckenmiller et al., 2020). The material from the Pardonet Formation such as the partial dentaries show a similar crown size and tooth density along the jaw margin as observed in *G. joseeae*. If proportionately comparable, these indicate an even smaller body size of roughly 1 m. Towards the end of the Triassic, thalattosaurs may display relatively reduced morphological disparity. Whether this pattern is genuine and reflects similar trends as seen in contemporaneous ichthyosaur disparity (Moon & Stubbs, 2020; Thorne et al., 2011), and thus hint at specific selective pressures, specialization in a few ecological niches, or selective extinction, remains unknown. To date, insufficient thalattosaur remains have been recovered from Norian deposits to unequivocally determine their diversity and disparity. The mandibular fragments and two partial skeletons (ROM, Toronto, Canada, temporarily not available to the author) from the Pardonet Formation remain undescribed and may provide crucial insight in the thalattosaur fauna of British Columbia during the Late Triassic and its affinity to contemporaneous forms such as *Gunakadeit joseeae*.

*G. joseeae* is a relatively small (75–90 cm) early diverging thalattosauroid from the Hound Island Volcanics (middle Norian, Alaska) (Druckenmiller et al., 2020). Additional remains from the middle Norian of the Keku Strait region include 19 remains of indeterminate claraziids (cf. *Nectosaurus* sp.), making it the most abundant occurrence from the Hound Island deposits (Adams, 2009). The Hound Island Volcanics material represents some of the youngest thalattosaurian remains worldwide (Druckenmiller et al., 2020). *G. joseeae* shows close affinity to Spathian-Middle Triassic taxa from British Columbia (*Thalattosaurus borealis* and *Paralonectes*) and the latest Anisian European thalattosauroids *Hescheleria* and *Clarazia* (Druckenmiller et al., 2020; Jiang et al., 2023). These close relationships require a ghost lineage that equals or exceeds roughly 20 million years and indicate that thalattosauroids acquired aquatic adaptations very early in their evolutionary history, allowing for trans-hemispheric dispersal (Druckenmiller et al., 2020). Druckenmiller et al., (2020) further postulates the likelihood that multiple cross-Panthalassan dispersal events have taken place throughout thalattosauroid evolution.

Another small (~ 1 m) early diverging thalattosaur from the Norian is the askeptosauroid *Endennasaurus acutirostris* from the Zorzino Limestone near Zogno, Lombardy, northern Italy (Müller, 2002; Müller et al., 2005; Paganoni & Pandolfi, 1989; Renesto, 1984, 1992, 2005). As is the case for *Gunakadeit*, the ghost lineage of the common ancestor of *Askeptosaurus italicus* and *Endennasaurus acutirostris* is also extensive, spanning at least 25 million years, between the upper Norian and latest Anisian. This too likely indicates a substantially lacking (northwestern) Tethyan fossil record for askeptosauroid material.

The Kössen Formation thalattosauroid from the late Norian-early Rhaetian of the northern Calcareous Alps of Austria, inhabited a shallow lagoonal or intrashelf basin along the northwestern margin of the Tethys (Müller, 2007). The fauna has a similar composition to that of the slightly older Zorzino Limestone (Müller, 2007). This relatively small (~ 1 m) thalattosauroid is often excluded from phylogenetic analyses due to its incomplete nature, however, it may be closely related to *Xinpusaurus* and/or *Nectosaurus* (Müller, 2007). This may again indicate a trans-Pacific migration prior to the late Ladinian-early Carnian, and the emergence of *Xinpusaurus* and *Nectosaurus,* and thus a substantial ghost lineage of almost 30 million years (Müller, 2007). It may also hint at multiple re-invasions of the western Tethys given the young age of the Kössen specimen and the lack of affinity with other western Tethyan taxa (Müller, 2002, 2007). The Kössen specimen represents the youngest definitive thalattosaur worldwide and indicates their presence up to or into the early Rhaetian.

The enigmatic *Pachystropheus* may have survived into the late Norian or early Rhaetian (Huene, 1935; Storrs, 1992, 1994; Storrs & Gower, 1993; Storrs et al., 1996; Renesto, 2005; Renesto & Dalla Vecchia, 2017; Čerňanský et al., 2020; Moreau et al., 2021; Cawthorne et al., 2024; Quinn et al., 2024). Whether these reports of putative Rhaetian thalattosaurs such as *Pachystropheus rhaeticus* and *Pachystropheus* sp. from Germany and the UK, as well as a potential *Endennasaurus*-like femur from the Fatra Formation of Slovakia represent true thalattosaur occurrences remains to be seen (Huene, 1935; Storrs, 1992, 1994; Storrs & Gower, 1993; Storrs et al., 1996; Renesto, 2005; Renesto & Dalla Vecchia, 2017; Čerňanský et al., 2020; Moreau et al., 2021; Cawthorne et al., 2024; Quinn et al., 2024).

Thalattosaurs may have gone extinct prior to the Triassic-Jurassic boundary, as has been suggested for various non-plesiosaurian sauropterygians, non-parvipelvian ichthyosaurs and marine archosauromorphs (Renesto & Dalla Vecchia, 2017; Gere et al., 2020). However, intensive sampling is needed to clarify their diversity patterns during the latest Triassic.

#### Biogeography

A complex biogeographic history involving periodic connectivity between the various northern hemispheric faunal provinces has been repeatedly hypothesized for thalattosaurs. A proposed close affinity of *Thalattosaurus* with the “claraziids” seems to indicate potential faunal connectivity between the eastern Panthalassic and the western Tethyan provinces, while the sister taxon relationship of some analyses between *Nectosaurus* and *Xinpusaurus* may illustrate potential trans-Pacific connectivity involving the eastern Tethyan faunal province (Jiang et al., 2004; Liu & Rieppel, 2001; Müller, 2002, 2005, 2007; Rieppel, 2019; Wu et al., 2009). Inclusion of *Anshunsaurus*, *Miodentosaurus*, and *Endennasaurus*, within the Askeptosauridae and Askeptosauroidea, respectively, illustrates a similar closeness between the eastern and western Tethyan faunas (Cheng et al., 2011; Müller, 2005; Rieppel, 2019; Wu et al., 2009).

The origin area for the Thalattosauriformes is difficult to determine, as the eastern and western Tethys and eastern Pacific provinces all are equally plausible or more accurately, equally unlikely. All these interpretations require extensive ghost-lineages and early widespread dispersal, such as for the origin of askeptosauroids and the last common ancestor of *Gunakadeit* and all other thalattosauroids (Druckenmiller et al., 2020; Müller, 2002). This is all indicative of a heavily undersampled, poor quality and biased thalattosaurian fossil record (Benson & Butler, 2011; Druckenmiller et al., 2020).

The earliest branching askeptosauroids in most analyses are *Askeptosaurus italicus* and *Endennasaurus acutirostris* which may hint at a western Tethyan origin of the clade and a dispersal into the Peritethys and eastern Tethyan realm during the latest Anisian (Druckenmiller et al., 2020; Müller, 2002, 2005). For thalattosauroids, the early diverging taxa in Druckenmiller et al., (2020) and Jiang et al., (2023) all represent North American forms (*Gunakadeit joseeae, Thalattosaurus, Nectosaurus*) which may be indicative of an eastern Panthalassic origin and subsequent trans-Panthalassan dispersal into the eastern Tethys. However, the current lack of thalattosaurs in the pre-Ladinian deposits of Chaohu, Luoping and Panxian is difficult to reconcile with this hypothesis. Furthermore, cranial similarities between *Nectosaurus* and *Xinpusaurus* may further hint at trans-Pacific dispersal events during the Ladinian and early Carnian (Liu & Rieppel, 2001; Müller, 2002; Pers. obs.).

Faunal similarities are also seen in Early Triassic ichthyosaurs between the eastern Tethys, eastern Panthalassa and high latitudes (e.g. Svalbard), before achieving a broadly cosmopolitan distribution at least by the Anisian (Ji et al., 2016; Lu et al., 2018; Rieppel, 2019). Non-plesiosaurian eosauropterygians seem to be largely restricted to the Tethys (though they dispersed across the Tethys multiple times), apart from pistosaurs or other similar medium-large-bodied forms which are sporadically but persistently present in the eastern Panthalassa as early as the Spathian (Bardet et al., 2014; Neenan et al., 2013; Sander et al., 1994, 1997; Storrs, 1991a, 1991b). Thalattosaurs show a somewhat intermediate pattern with askeptosauroids being exclusively Tethyan in distribution, while the potentially more highly adapted coastal or even coastal pelagic aquatic thalattosauroids spread across the Tethyan and eastern Panathalassic Provinces (Merriam, 1904, 1905, 1907, 1908; Storrs, 1991a, 1991b; Nicholls & Brinkman, 1993; Sander et al., 1994, 1997; Nicholls, 1999; Rieppel et al., 2000; Sues & Clark, 2005; Neenan et al., 2013; Bardet et al., 2014; Metz et al., 2016; Metz, 2019; Druckenmiller et al., 2020).

Overall, the earliest thalattosaurs may have been restricted to the north(west)ern Pangean coastline due to the lack of intracontinental seaways, leaving just the peri-Pangean, Tethyan and trans-Panthalassan dispersal routes (Bardet et al., 2014; Ji et al., 2016; Lu et al., 2018; Rieppel, 2019). During the unstable and periodic warm climatic conditions of the Early Triassic ichthyosaurs, sauropterygians and thalattosaurs possibly dispersed via the high-latitude Arctic, via the Boreal route (Hallam, 1994 in: Bardet et al., 2014). Global transgression in the Middle Triassic facilitated dispersal throughout the Tethyan realm into the western Tethys (Bardet et al., 2014). During the Late Triassic, the shrinkage of habitats may have increased the vulnerability of thalattosaurs to environmental perturbations, resulting in their eventual demise.

More explorative work, as well as morphological and phylogenetic studies are required to unravel the paleobiogeography of thalattosaurs in more detail.

### Paleoecology of thalattosaurs

The two (super)families within Thalattosauriformes, the Askeptosauroidea and the Thalattosauroidea, show prominent differences in dental, cranial and skeletal morphology that may reflect different ecologies and lifestyles (Cheng et al., 2011; Druckenmiller et al., 2020; Jiang et al., 2023; Li et al., 2016; Liu & Rieppel, 2005; Liu et al., 2013; Müller, 2002, 2005, 2007; Wu et al., 2009).

#### Askeptosauroidea

Most askeptosauroids are characterized by well-developed robust limbs; the humerus often being slightly shorter than the femur; shorter zeugopodia than stylopodia; well ossified carpal and manus elements; and presumed paddle-like extremities adorned by sharp claws; all of which are indicative of at least some degree of terrestrial capabilities (Müller, 2002, 2005; Müller et al., 2005; Renesto, 1984, 1992; Rieppel, 2019). Paradoxically, the stronger gastral basket of askeptosauroids such as those of *Miodentosaurus* (Cheng et al., 2007a, 2007b; Wu et al., 2009) and *Endennasaurus* (Müller, 2002; Müller et al., 2005; Paganoni & Pandolfi, 1989; Renesto, 1984, 1992, 2005) would have made the trunk more rigid. This would have allowed for more energy efficient long-distance dispersal and potentially achievement of high swimming speeds, but likely merely reflects more involvement of the limbs during swimming and a larger terrestrially-oriented lifestyle (Benson & Butler, 2011; Müller, 2002; Müller et al., 2005; Naish, 2023). Synapomorphies include a straight dentigerous or edentulous rostrum, elongated necks with cervical vertebral counts exceeding ten, and lack of palatal dentition (Müller, 2002, 2005; Müller et al., 2005).

The skull of *Askeptosaurus italicus* is presumed to reflect the plesiomorphic state, with a separate prefrontal and lacrimal as well as a postfrontal and postorbital bone, in addition to the presumed presence of a slit-like upper temporal fenestra and thus diapsid affinities (Müller, 2005; Rieppel et al., 2005). The dentition of most askeptosauroids differs from that of thalattosauroids in being relatively isodont, occupying a large continuous extent of the jaw margins, sharp conical and often slightly recurved. This may suggest a more generalist diet of small to medium-sized vertebrates and perhaps cephalopods (Müller, 2002). The flattened cranium and long flexible but powerful neck were likely well-suited for lateral snapping bites (Müller, 2002; Rieppel, 2019; Rieppel et al., 2005). *Endennasaurus* with its edentulous jaws likely would have fed on soft small prey, such as soft-shelled invertebrates, crustaceans and occasionally small vertebrates such as fish (Müller, 2002; Müller et al., 2005; Naish, 2023). *Miodentosaurus brevis* (Cheng et al., 2007a, 2007b) displays a highly divergent morphology compared to other askeptosauroids, seemingly convergent with thalattosauroids. *Miodentosaurus* has a short slightly blunted and lightly ventrally-curving premaxillary rostrum, and reduced dental occupation with teeth only present in the premaxilla and the anterior dentary (Cheng et al., 2007a, 2007b). The dental (Pierce II of Benton et al., 2013) and rostral morphology similar to that of *Xinpusaurus* and *Thalattosaurus*, respectively, may hint at a diet likely consisting of fish or cephalopods, however, the large body size makes it more likely to be rather opportunistic or generalistic. During the late Ladinian and Carnian, highly nested askeptosauroids such as *Anshunsaurus*, seem to become more adapted to the aquatic environment, as illustrated by a very long laterally compressed tail and highly reduced limbs (Liu & Rieppel, 2005; Maisch, 2015; Naisch, 2023).

#### Thalattosauroidea

The Thalattosauroidea typically display a higher degree of morphological heterogeneity in dental and rostral shapes, illustrated by the moderate to strong ventral deflection of the rostrum and the high degree of heterodonty, which may indicate a much broader ecological niche space occupation (Bardet et al., 2014; Benton et al., 2013; Liu & Rieppel, 2001; Muller, 2005; Rieppel et al., 2005). Additionally, they differ from askeptosauroids in that they have a shorter neck with some having as few as 4 cervical vertebrae (e.g. Druckenmiller et al., 2020; Liu et al., 2013; Zhao et al., 2013). Other differences include divergent limb proportions, being short and stout, and poorly ossified carpal and tarsal bones, that may reflect a more exclusively aquatic lifestyle (Benton, 2005; Chai & Jiang, 2021; Druckenmiller et al., 2020; Liu et al., 2013; Naish, 2023; Zhao et al., 2013). Nonetheless, lateral undulation of the body axis was still likely the main source of propulsion (Liu et al., 2013; Naish, 2023; Zhao et al., 2013). Their aquatic capabilities are also indicated by the widespread distribution of thalattosauroids across the northern hemisphere very early in their evolutionary history (Bastiaans et al., 2023). Similarly, the reduction of number of cervical vertebrae in thalattosauroids may be a further indication of greater marine adaptations, as mosasaurs, ichthyosaurs, pliosaurs and cetaceans display a similar trend (Wade, 1984; O’Keefe, 2001, 2002; Spoor et al., 2002; Lindgren et al., 2007). This may be linked to decreased neck mobility resulting in efficient gaze and body stabilization and related decreasing sensitivity of the vestibular system (Wade, 1984; O’Keefe, 2001, 2002; Spoor et al., 2002; Benton, 2005; Lindgren et al., 2007). Later thalattosauroids (e.g. *Gunakadeit*, Brisbois Mb taxon) also show a shortening of the posterior skull region as observed in advanced marine ichthyosaurs (Benton, 2005; Druckenmiller et al., 2020; Metz, 2019).

##### Rostral morphologies

Rostral shapes in thalattosauroids can be broadly subdivided into three general categories: (I) straight pointed rostra (e.g. *Gunakadeit*) (Druckenmiller et al., 2020); (II) slightly elongate with little to modest ventral deflection, and in many with a distinct diastema between premaxillary and maxillary dentition (e.g. *Xinpusaurus*, *Thalattosaurus*, and *Clarazia*); (III) the most extreme forms of these curved premaxillary rostra are near vertically- or even slightly posteroventrally-oriented, with short dentigerous margins separated by a distinct diastema from the other marginal teeth of the upper jaw (e.g. *Hescheleria*, *Nectosaurus*, XNGM WS-22-R5, the Natchez Pass claraziid, the Brisbois mb thalattosaur and possibly *Paralonectes*) and mandibles shorter than their upper jaws (Peyer, 1936a, 1936b; Rieppel, 1987; Storrs, 1991b, Nicholls & Brinkman, 1993; H.-D., Sues Pers. Comm.; Rieppel et al., 2005; Sues & Clark, 2005; Metz, 2019; Chai et al., 2020a, 2020b). Generally, thalattosauroids show decreased tooth counts and limited distribution of teeth along the margins of the upper and lower jaws relative to askeptosauroids. Furthermore, thalattosauroids have a prominent subnarial process of the premaxilla (e.g. TMP 1996.72.1; Merriam, 1905; Nicholls, 1999; Rieppel & Liu, 2006; Metz, 2019) which is lacking in askeptosauroids (Cheng et al., 2007a, 2007b; Kuhn, 1952; Müller, 2005; Müller et al., 2005). *Hescheleria ruebeli* has an additional unusual feature, namely a bony cusp on the symphyseal region of the lower jaw that far exceeds the surrounding mandibular dentition in height. This structure likely could have, in concert with the ventral premaxilla or vomerine, served to crush (hard-)shelled invertebrate prey (Peyer, 1936b; Rieppel, 2019; Rieppel et al., 2005). The rostral shapes of thalattosaurs are likely indicative of specific feeding ecology or dietary specializations, such as probing muddy shelf sediments or being very streamlined fast burst or ambush predators. However, these hypotheses regarding the significance and functionality of the ventrally deflecting premaxillae in derived thalattosauroids have not been tested and thus their use remains a mystery (Peyer, 1936a, 1936b; Rieppel, 1987). Whether the strongly deflected rostral morphologies are plesiomorphic for thalattosauroids, and secondarily lost in certain taxa, thus indicative for a close relationship between *Nectosaurus*, *Hescheleria*, *Paralonectes,* the Natchez Pass claraziid, the Brisbois Mb thalattosaur and XNGM WS-22-R5 or whether these are autapomorphic and evolved independently numerous times in Thalattosauria needs further testing through detailed phylogenetic analyses (Chai et al., 2020a, 2020b; Liu & Rieppel, 2001; Metz, 2019; Nicholls, 1999; Peyer, 1936b; Rieppel et al., 2005; Sues & Clark, 2005). Rieppel et al (2005), as opposed to Liu and Rieppel (2001), suggested that the dorsal curvature of the anterior alveolar margin is not a synapomorphy for *Xinpusaurus*, *Nectosaurus* and *Paralonectes* but instead represent an autapomorphy of *Xinpusaurus*, thus suggesting an independent evolution of the associated ventrally deflecting premaxillae.

##### Durophagy

Durophagous marine reptiles constitute important components of Middle and early Late Triassic ecosystems as illustrated by their high taxic diversity and abundance (Crofts et al., 2015, 2017; Schmitz et al., 2005). Many thalattosauroids display bulbous or flattened tooth crowns on the marginal or palatal elements combined with deep heavily built mandibles, and variable degrees of rostral deflection. Often it is assumed to be indicative of a diet of epi- and endobenthic (hard-)shelled invertebrate prey such as bivalves and perhaps cephalopods (ammonoids and belemnoids) (Muller, 2002; Rieppel, 2019). In fact, Lower-Middle Triassic thalattosauroids (e.g. *Paralonectes, Agkistrognathus,* TMP 1989.127.18) already display a high degree of dental disparity and heterodonty, low tooth counts, and even medially migrating posterior mandibular teeth (Bastiaans et al., 2023). The only clear exceptions within thalattosauroids being the mandibular dentition of *Nectosaurus halius*, the Brisbois Member thalattosaur and *Gunakadeit* which are lacking molariform posterior mandible teeth (Druckenmiller et al., 2020; Merriam, 1905, 1908; Metz, 2019; Nicholls, 1999). The presence of durophagous dentition is mutually exclusive with strong pelagic capabilities (Kelley et al., 2014). It is thus likely that thalattosauroids inhabited the shallow marginal seas but may have become increasingly coastal-pelagically adapted with heterodont dentition that allowed for occupation of various palaeoecological guilds. Askeptosaurs, on the other hand, were likely more near-surface water generalists (Bardet et al., 2014; Benton et al., 2013; Chai & Jiang, 2021; Müller, 2002; Rieppel, 2019). Other taxa, such as *Xinpusaurus* for instance are superficially ichthyosaur-like, with long slender serpentine bodies and snouts, relatively small heads, and often found associated with ammonoids and fishes (Benton et al., 2013; Lu et al., 2018). Ammonoids likely composed an important food source for many thalattosauroids, however, their heterodont dentition likely allowed them to exploit a wide variety of prey items (Benton et al., 2013; Druckenmiller et al., 2020; Merriam, 1905; Müller, 2002; Nicholls, 1999). Gut contents devoid of shells, bones and scales have been found in *Gunakadeit*, indicating inclusion of soft bodied prey into their diets and specialized hyoid apparatuses that may have supported suction feeding or enhanced chemosensing (Druckenmiller et al., 2020).

Overall, thalattosaurs likely occupied various ecological niches with a wide array of feeding styles, including shallow marine predators of endo- and epibenthic shelled invertebrate prey, small, medium and large-sized ambush predators with smashing, crunching, crushing and piercing (Pierce I & II type) dentitions (Merriam, 1905; Peyer, 1936a, 1936b; Rieppel, 1987; Nicholls, 1999; Benton et al., 2013: 234; Chai et al., 2020a, 2020b). In the Carnian thalattosaurs seemingly occupy some overlapping feeding guilds as ichthyosaurs of Anisian and perhaps Ladinian ecosystems (e.g. Smash, Crunch, Crush), while the Norian taxa with simplified piercing dentition may perhaps resemble later more coastal-pelagic ichthyosaurs in lifestyle and diet (Benton et al., 2013, p. 226).

## Preliminary conclusions and future directions

There are a multitude of reasons that the Thalattosauriformes should be revised and restudied. (I) They can potentially serve to illustrate the morphological transitions during the earliest adaptive phases of the evolution of secondarily aquatic marine reptiles. The combination of taxa with potentially plesiomorphic morphologies with presumed “terrestrial signatures”, displaying modest trunks with elongate limbs and tails, and seemingly minor to moderately adapted aquatic taxa with relatively longer trunks, shorter limbs and shorter necks make them particularly interesting (as suggested for the potentially early diverging sauropterygiform *Hanosaurus* by Wang et al., 2022). (II) Furthermore, shallow marine habitats display a greater degree of endemic fauna with higher turnover rates, especially for large durophagous predators, making them particularly interesting for macroevolutionary research and documenting small abiotic changes (Kelley et al., 2014; Scheyer et al., 2014; Kelley & Pyenson, 2015). Thalattosaurs can thus serve as a paradigm for rapid morphological and presumed functional diversification after the EPME and their high disparity reflective of exploitation of unsampled niche space for marine amniotes. (III) Lastly, thalattosaurs may represent early diverging (neo)diapsids, as postulated based on their temporal architecture, and could therefore be of considerable interest for understanding early (neo)diapsid evolution.

A detailed review of thalattosaur research shows that thalattosaurs may represent more common faunal components in Triassic ecosystems than previously assumed. However, their record is heavily influenced by Lagerstätten effects as well as incomplete sampling of thalattosaur-bearing localities such as those of the Lower Triassic of British Columbia and the Upper Triassic of the Alpine region. Thalattosaurs, like ichthyosaurs and sauropterygians, most likely rapidly radiated in the late Early and early Middle Triassic, attaining a northern hemispheric distribution prior to the late Middle Triassic. New discoveries in recent years have underscored the high degree of morphological disparity of the group, including rostral shapes, dentition types and skeletal proportions, which likely reflect different ecologies and lifestyles. However, much is still unclear about the phylogenetic position of thalattosaurs within Diapsida and their relationships to other Mesozoic marine reptile groups such as sauropterygians and ichthyopterygians. Also, the interrelationships between the various thalattosaur species are still poorly resolved as a result of the lack of detailed morphological knowledge for most taxa. Research on the Thalattosauriformes remains heavily centered on morphological descriptive work of well-preserved specimens from Lagerstätten and highly fossiliferous localities, such as those from western North America, central Europe and southwestern China. This leaves a wealth of unexploited research potential for future scientific endeavors that should try to build on the strong basis of nearly a century of morphological work, such as that on the famous Monte San Giorgio deposits (e.g. Peyer, 1936a, 1936b; Kuhn-Schnyder, 1952; Rieppel, 1987; Müller et al., 2005). The recent influx of new and well-preserved three-dimensional thalattosaur material from southwestern China and North America provides a unique opportunity to study various aspects of thalattosaur biology for the first time in a broader context. Several potential avenues include:

### 3D cranial morphology

The usage of innovative 3D methodology, such as state-of-the-art digital visualizations and three-dimensional reconstructions, is becoming more commonplace in paleontology, allowing researchers to study taxa in unprecedented detail. However, the effectiveness and fidelity of conventional radiographic approaches is determined by the nature of preservation, therefore considerable focus should lie on finding alternative high resolution scanning methods for studying the fragmentary and highly problematic thalattosauriform remains. This would maximize data extraction from historically problematic, and thus largely ignored, fossil material, and will provide glimpses into the 3D anatomy of this small but presumably ecomorphologically diverse group of animals.

### Taxonomic data & phylogeny

The newly acquired morphological data will aid much needed future taxonomic and systematic studies on diapsid and thalattosaur ingroup relationships. Despite prominent recent improvements, phylogenetic analyses are still limited by low character counts and morphological uncertainty. Modern revisions of historic taxa and the wealth of new specimens will solve many of the current problems and will further help address issues such as ontogenetic synonyms (Chai & Jiang, 2021; Chai et al., 2023; He et al., 2023; Liu, 2013; Maisch, 2014; Rieppel & Liu, 2006; Wu et al., 2009) and contested taxa; and the presence or absence of an upper temporal fenestra. In turn this may lead to a better understanding of the phylogenetic placement of thalattosaurs whether within or outside of Diapsida as well as the interrelationships within Thalattosauriformes.

### Biases

A stable well-informed phylogeny for thalattosaurs will provide a framework for studies on geological megabiases and the effects of heterogeneous sampling efforts on both the group itself and marine reptiles cumulatively. This is important to quantify before being able to disseminate their evolutionary history, biodiversity and paleobiogeography through time. Although large scale patterns in marine tetrapod diversity during the Triassic can be reliably reconstructed (Benson et al., 2010), it is vital to quantify the influence of Lagerstätten and other exceptional fossiliferous localities on the available phylogenetic information and biodiversity trends in marine reptiles, especially for clades such as thalattosaurs (Benson & Butler, 2011; Benson et al., 2010; Kelley & Pyenson, 2015; Woolley et al., 2024). This may inform on whether or not thalattosaur evolution can be explained through an early burst model, thus reflecting similarities to ichthyosaurian evolution, whereby initial high evolutionary rates and high morphological diversity gave way to lower rates and lower disparity (e.g. Moon & Stubbs, 2020).

### Ecology and functional biology

The observable high morphological disparity in body sizes, rostral shapes and dentition types, has raised numerous questions about their feeding mechanics and ecological niche fill that have yet to be adequately addressed. Focal points should be:

### Early aquatic adaptations and the marine transition

Thalattosaurs seemingly display a continuum of aquatic adaptations ranging from presumed largely terrestrial early diverging askeptosaurs to perhaps coastal-pelagic highly nested thalattosauroids. Given the repeated evolution of similar adaptations and modifications of the appendicular and axial skeleton in marine reptile clades it is likely that similar developmental or perhaps genetic pathways paved the way for similar land-sea transitions in other marine reptile clades (Kelley & Pyenson, 2015). The earliest diverging representatives of thalattosaurs may thus elucidate the plesiomorphic morphologies associated with the initial phases of morphological adaptive evolution associated with the transition from terrestrial to aquatic lifestyles.

### Shape and functional analyses

Quantitative shape analyses of cranial, dental and postcranial morphology may further aid in assigning thalattosaur taxa to hypothetical feeding guilds, establish differential ecomorphospace occupations or detail changes in disparity through time (e.g. Fischer et al., 2022; Massare, 1987; Moon & Stubbs, 2020; Stubbs & Benton, 2016; Wang et al., 2022). These analyses, however, need to be done in concert with morphofunctional analyses such as FEA or MDA, as shape alone does not necessarily accurately reflect functionality (e.g. Kelley & Pyenson, 2015; Lautenschlager et al., 2016). In fact, a multiproxy approach is needed to accurately reflect diet and trophic interactions in extinct taxa, including methods such as 3D geometric morphometrics, FEA or MDA, dental microwear analyses as well as direct observations of stomach contents (e.g. Jiang et al., 2020; Kear et al., 2003). This will allow us to address topics such as: their place in Triassic foodwebs, and hypotheses regarding their extinction, such as reduced disparity and superficial convergence with ichthyosaurs towards the latest Triassic.

### Histology and aquatic adaptations

Additional histological work is required to detail bone microanatomy as well as shed light on the growth rates and life history traits of thalattosaurs. Considerable work has been done on other marine reptile groups such as mosasaurs (Houssaye et al., 2013; Pellegrini, 2007), ichthyosaurs (Houssaye et al., 2014, 2018; Kolb et al., 2011; Nakajima et al., 2014), sauropterygians (Hugi, 2011; Klein, 2010; Klein et al., 2015a, 2015b, 2016; Krahl et al., 2013; Sander & Wintrich, 2021) and tanystropheids (Jaquier & Scheyer, 2017; Spiekman et al., 2020). Only recently the first histological examination of thalattosaur remains has been conducted (Klein et al., 2023). This prompted interesting results regarding differences in growth rates, lifestyles and potential degree of aquatic adaptation between thalattosauroid and askeptosauroid thalattosaurs. Larger-scale histological examinations including more taxa may help address biogeographical issues by informing on the aquatic capabilities of thalattosaurs and thus the plausible dispersal routes.

### Endocranial work

The availability of three-dimensionally preserved crania for several taxa may open up other avenues to explore the degree of aquatic adaptation of thalattosaurs, for instance by detailed endocranial comparison. The neuroanatomy of other marine reptile groups such as tanystropheids (e.g. Spiekman et al., 2020), sauropterygians (e.g. Allemand et al., 2022; Neenan & Scheyer, 2012; Neenan et al., 2017; Voeten et al., 2018), and ichthyosaurs (e.g. Allemand et al., 2022; Marek et al., 2015) have recently received considerable attention. The morphology of the endosseous labyrinth may reflect a degree of aquatic specialization such as swimming capabilities and be associated with postcranial skeletal adaptations (especially neck length) (Neenan et al., 2017). The availability of several well-preserved three-dimensional braincases for thalattosaurs including the Brisbois Mb. thalattosaur, *Xinpusaurus suni*, *Anshunsaurus huangguoshuensis*, and *Miodentosaurus brevis* may offer the first insights into the endocranial morphology of thalattosaurs as well as hint at neurosensory adaptations.

Despite over a century of research, very little is known about the enigmatic Thalattosauriformes. Technological advances and the addition of a wealth of new material allow us to re-analyze historically important material. These advances will continue to add to the long list of important discoveries made at important Lagerstätten, such as those of Monte San Giorgio.

## Data Availability

This review is based on previously published material and personally observed material in various collections across North America and Europe. References to published data are given throughout the text, in Table

**Table 1:** Comprehensive overview of confirmed and postulated thalattosauriform-bearing formations and information on repositories containing thalattosaurian material

Name	Locations	Formations	Ages	Repository	Source
Thlattosauridae cf. *Paralonectes*	Meosin Mountain, British Columbia, Canada	Meosin Mountain Fm	Smithian? (Olenekian)?	Royal Tyrrell Museum of Paleontology, Alberta, Canada	–
Thalattosauridae indet	Meosin Mountain, British Columbia, Canada	Meosin Mountain Fm	Smithian? (Olenekian)?	Royal Tyrrell Museum of Paleontology, Alberta, Canada	–
Thalattosauridae cf. *Agkistrognathus*/*Paralonectes*	Meosin Mountain, British Columbia, Canada	Meosin Mountain Fm	Smithian? (Olenekian)?	Royal Tyrrell Museum of Paleontology, Alberta, Canada	–
*Wapitisaurus problematicus*	Wapiti Lake, Cirque B, British Columbia, Canada	Sulphur Mountain Fm	Smithian? (Olenekian)?	Royal Tyrrell Museum of Paleontology, Alberta, Canada	Brinkman (1988); Bastiaans et al., (2023)
Thalattosauridae indet (cf. *Agkistrognathus*/*Paralonectes*)	Wapiti Lake, Cirque N, British Columbia, Canada	Sulphur Mountain Fm	Olenekian?	Royal Tyrrell Museum of Paleontology, Alberta, Canada	–
*Agkistrognathus campbelli*	Wapiti Lake, Cirque D, British Columbia, Canada	Sulphur Mountain Fm	Lower- Middle Triassic	Royal Tyrrell Museum of Paleontology, Alberta, Canada	Nicholls and Brinkman (1993)
*Paralonectes merriami*	Wapiti Lake, Cirque D, British Columbia, Canada	Sulphur Mountain Fm	Lower- Middle Triassic	Royal Tyrrell Museum of Paleontology, Alberta, Canada	Nicholls and Brinkman (1993)
Thalattosauridae cf. *Thalattosaurus*	Wapiti Lake, Cirque D, British Columbia, Canada	Sulphur Mountain Fm	Lower- Middle Triassic	Royal Tyrrell Museum of Paleontology, Alberta, Canada	–
Thalattosauridae indet	Wapiti Lake, Cirque D, British Columbia, Canada	Sulphur Mountain Fm	Lower- Middle Triassic	Royal Tyrrell Museum of Paleontology, Alberta, Canada	Nicholls and Brinkman (1993)
cf. Thalattosauriformes	Augusta Mountains, Nevada, USA	Favret Fm	Anisian (Middle Triassic)	Field Museum of Natural History, Chicago, USA	Sander et al., (1994)
*Askeptosaurus italicus*	Canton Ticino, Switzerland; Varese Province, Italy	Besano Fm	Anisian (Middle Triassic)	Paleontological Institute, Zurich, Switzerland; Museo di Scienze Naturali Milano, Italy	Nopcsa (1925); Kuhn (1952); Müller (2005)
*Clarazia schinzi*	Canton Ticino, Switzerland	Besano Fm	Anisian (Middle Triassic)	Paleontological Institute, Zurich, Switzerland	Peyer (1936a); Rieppel (1987)
*Hescheleria ruebeli*	Canton Ticino, Switzerland	Besano Fm	Anisian (Middle Triassic)	Paleontological Institute, Zurich, Switzerland	Peyer (1936b); Rieppel (1987)
Thalattosauridae cf. *Paralonectes*/*Thalattosaurus perrini*	Wapiti Lake, Cirque T, British Columbia, Canada	Sulphur Mountain Fm	Late Anisian-lower Ladinian ( Middle Triassic)	Royal Tyrrell Museum of Paleontology, Alberta, Canada	Nicholls and Brinkman (1993)
*Thalattosaurus borealis*	Wapiti Lake, Cirque T, British Columbia, Canada	Sulphur Mountain Fm	Late Anisian-lower Ladinian ( Middle Triassic)	Royal Tyrrell Museum of Paleontology, Alberta, Canada	Nicholls and Brinkman (1993)
*Blezingeria ichthyospondylus*	Germanic Basin, Southwestern Germany	Upper Muschelkalk & lower Lettenkeuper	Ladinian (Middle Triassic)	Geologisch-Paläontologisches Institut der Universität Tubingen; Muschelkalk Museum Hagdorn Ingelfingen; Staatliches Museum für Naturkunde Stuttgart, Germany	Fraas (1896); Müller (2002); Diedrich (2015); Schoch (2015)
*Xinpusaurus xingyiensis*	Xingyi City, Guizhou Province, China	Falang Formation	Ladinian (Middle Triassic)	Xingyi National Geopark Museum, Xingyi, Guizhou Province, China	Li et al., (2016)
XNGM-WS-22-R5	Wusha Town, Xingyi, Guizhou Province, China	Falang Formation	Ladinian (Middle Triassic)	Xingyi National Geopark Museum, Xingyi, Guizhou Province, China	Chai et al., (2020b)
*Anshunsaurus* cf. *A. huangguoshuensis*	Xingyi, Guizhou Province, China	Falang Formation	Ladinian (Middle Triassic)	Xingyi National Geopark Museum, Xingyi, Guizhou Province, China	Chai et al., (2020a)
Thalattosauridae indet	Between Mont-ral & Alcover, Tarragona, Spain	Spanish Muschelkalk	Upper Ladinian (Middle Triassic)	Museo y Laboratoria de Geologia, Seminario de Barcelona, Spain	Rieppel and Hagdorn (1998)
*Anshunsaurus huangnihensis*	Near Xingyi, Guizhou Province, China	Falang Fm. (Zhuganpo Fm.)	Upper Ladinian (Middle Triassic)	Yichang Institute of Geology and Mineral Resources, Hubei, China	Cheng (2007a); Cheng et al., (2011)
*Anshunsaurus wushaensis*	Near Xingyi, Guizhou Province, China	Falang Fm. (Zhuganpo Fm.)	Upper Ladinian (Middle Triassic)	Institute of Vertebrate Paleontology and Paleoanthropology, Beijing, China	Rieppel et al., (2006)
Thalattosauriformes cf. *Askeptosaurus*	Luoping County, Yunnan Province, China	Gejiu Fm	Upper Ladinian (Middle Triassic)	Geological Museum, Peking University, Beijing, China	Sun et al., (2005)
Natchez Pass taxon	Humboldt County, Nevada, USA	Natchez Pass Fm	Lower Carnian (Upper Triassic)	Smithsonian National Museum of Natural History, Washington DC, USA	Storrs (1991b); Sues and Clark (2005)
Thalattosauriformes cf. *Endennasaurus*	Julian Alps, Italy	Predil Limestone Fm	Lower Carnian (Upper Triassic)	Museum Friulano di Storia Naturale, Udine	Dalla Vecchia (1993)
*Xinpusaurus suni*	Guanling County, Guizhou Province, China	Xiaowa Fm	Lower Carnian (Upper Triassic)	Institute of Vertebrate Paleontology and Paleoanthropology, Beijing, China	Yin et al., (2000); Rieppel and Liu (2006; Liu et al., 2013)
*Thalattosaurus alexandrae*	Shasta County, California, USA	Hosselkus Limestone Fm	Upper Carnian (Upper Triassic)	University of California Museum of Paleontology, Berkeley, California, USA; United States National Museum, Smithsonian Institution, Washington DC, USA	Merriam (1904, 1905); Nicholls (1999)
*Thalattosaurus perrini*	Shasta County, California, USA	Hosselkus Limestone Fm	UPPER Carnian (Upper Triassic)	California Academy of Sciences, California, USA	Merriam (1904, 1905)
*Thalattosaurus* sp.	Shasta County, California, USA	Hosselkus Limestone Fm	Upper Carnian (Upper Triassic)	Sierra College, Rocklin, California, USA	–
*Thalattosaurus shastensis* (Thalattosauridae cf. *Nectosaurus*)	Shasta County, California, USA	Hosselkus Limestone Fm	Upper Carnian (Upper Triassic)	University of California Museum of Paleontology, Berkeley, California, USA	Merriam (1905); Nicholls (1999)
*Nectosaurus halius*	Shasta County, California, USA	Hosselkus Limestone Fm	Upper Carnian (Upper Triassic)	University of California Museum of Paleontology, Berkeley, California, USA	Merriam (1905, 1908); Nicholls (1999)
*Anshunsaurus huangguoshuensis*	Guanling County, Guizhou Province, China	Xiaowa Fm	lower Carnian (Upper Triassic)	Institute of Vertebrate Paleontology and Paleoanthropology, Beijing, China	Liu (1999); Rieppel et al., (2000); Liu and Rieppel (2005); Liu (2007); Maisch and Hao (2008); Maisch (2015)
*Concavispina biseridens*	Guanling County, Guizhou Province, China	Xiaowa Fm	lower Carnian (Upper Triassic)	Zhejiang Museum of Natural History, Hangzhou, Zhejiang, China	Zhao et al., (2013); Liu et al., (2013)
*Miodentosaurus brevis*	Guanling County, Guizhou Province, China	Xiaowa Fm.?	Lower? Carnian (Upper Triassic)	National Museum of Natural Science, Taiwan; Zhejiang Museum of Natural History, Hangzhou, Zhejiang, China	Cheng et al., (2007b); Wu et al., (2009); Zhao et al., (2010)
*Neosinasaurus hoangi*	Guanling County, Guizhou Province, China	Xiaowa Fm.?	Lower? Carnian (Upper Triassic)	Geological Survey and Research Institute, Guizhou Provincial Bureau of Geology and Mineral Resources, Guiyang, Guizhou, China	Yin et al., (2000)
*Wayaosaurus geei*	Guanling County, Guizhou Province, China	Xiaowa Fm.?	Lower? Carnian (Upper Triassic)	Geological Survey and Research Institute, Guizhou Provincial Bureau of Geology and Mineral Resources, Guiyang, Guizhou, China	Yin et al., (2000); Wu et al., (2009); Chai and Jiang (2021)
*Wayaosaurus bellus*	Guanling County, Guizhou Province, China	Xiaowa Fm.?	Lower? Carnian (Upper Triassic)	Geological Survey and Research Institute, Guizhou Provincial Bureau of Geology and Mineral Resources, Guiyang, Guizhou, China	Yin et al., (2000); Wu et al., (2009); Chai and Jiang (2021)
*Xinpusaurus bamaolinensis*	Guanling County, Guizhou Province, China	Xiaowa Fm.?	Lower? Carnian (Upper Triassic)	Yichang Institute of Geology and Mineral Resources, Hubei, China	Cheng (2003); Liu (2013); Li et al., (2016); Maisch (2014
*Xinpusaurus kohi*	Guanling County, Guizhou Province, China	Xiaowa Fm.?	Upper? Carnian (Upper Triassic)	Geological Museum, Peking University, Beijing, China	Jiang et al., (2004); Maisch (2014)
Brisbois Mb Taxon	Near Paulina, Oregon, USA	Vester Fm	Upper Carnian	University of Oregon Museum of Natural and Cultural History, Eugene, Oregon, USA	Metz et al. (2015, 2016, 2019)
Thalattosauriformes indet	Pink Mountain, British Columbia, Canada	Pardonet Fm	Norian	Royal Tyrrell Museum of Paleontology, Alberta, Canada	Storrs (1991b)
Thalattosauridae indet	Williston Lake, Jewitt Spur, Hudson’s Hope, British Columbia, Canada	Pardonet Fm	Norian	Royal Ontario Museum, Toronto, Ontario, Canada	–
*Endennasaurus acutirostris*	Endenna, Lombardy, Italy	Zorzino Limestone	Norian	Museo Civico di Scienze Naturali 'E. Caffi', Bergamo, Lombardy, Italy; Dipartimento di Scienze della Terra of Universita degli Studi di Milano	Renesto (1984, 2005); Paganoni and Pandolfi (1989); Müller et al., (2005); Renesto (2005)
*Gunakadeit joseeae*	Keku Islands of Southeast Alaska	Hound Island Volcanics	Middle Norian	University of Alaska Fairbanks, Alaska, USA	Druckenmiller et al., (2020)
Claraziidae indet. (Thalattosauridae cf. *Nectosaurus* sp.)	Hound Island, southeastern Alaska	Hound Island Volcanics	Middle Norian	southern methodist university?, Dallas, Texas, USA	Adams (2009)
Kössen Fm. taxon	Gaissau near Salzburg, Austria	Kössen Fm	Upper Norian-lower Rhaetian	Staatliches Museum für Naturkunde Stuttgart, Germany	Müller (2007)
Thalattosauriformes cf. *Endennasaurus*	Úbočka near Čičmany, Strážovské Mountains, Slovakia	Fatra Fm	Rhaetian	Slovak National Museum, Bratislava	Čerňanský et al., (2020)
Aff. *Pachystropheus* sp.	Aust Cliff, Gloucestershire, UK; Bonenburg, City of Warburg, Kreis Höxter, North Rhine-Westphalia, Germany	Westbury Fm.; “Rhaetian bone beds”	Rhaetian	Natural History Museum, London, UK; Bristol Museum and Art Gallery, Bristol, UK; University of Bristol Geology Collection, Bristol, U.K; LWL-Museum für Naturkunde, Münster, Germany	Huene (1935); Storrs (1992); Storrs and Gower (1993); Storrs et al., (1996); Renesto (2005); Quinn et al., (2024)
*Pachystropheus rhaeticus*	Emborough Quarry, Radstock BA3 4TZ, UK	Westbury Fm	Rhaetian	Natural History Museum, London, UK	Cawthorne et al., (2024)
*Pachystropheus rhaeticus*	Saltford, near Bath, SW UK	Westbury Fm	Rhaetian	Bristol Museum and Art Gallery, Bristol, UK	Moreau et al., (2021)

**Fig. 4:**
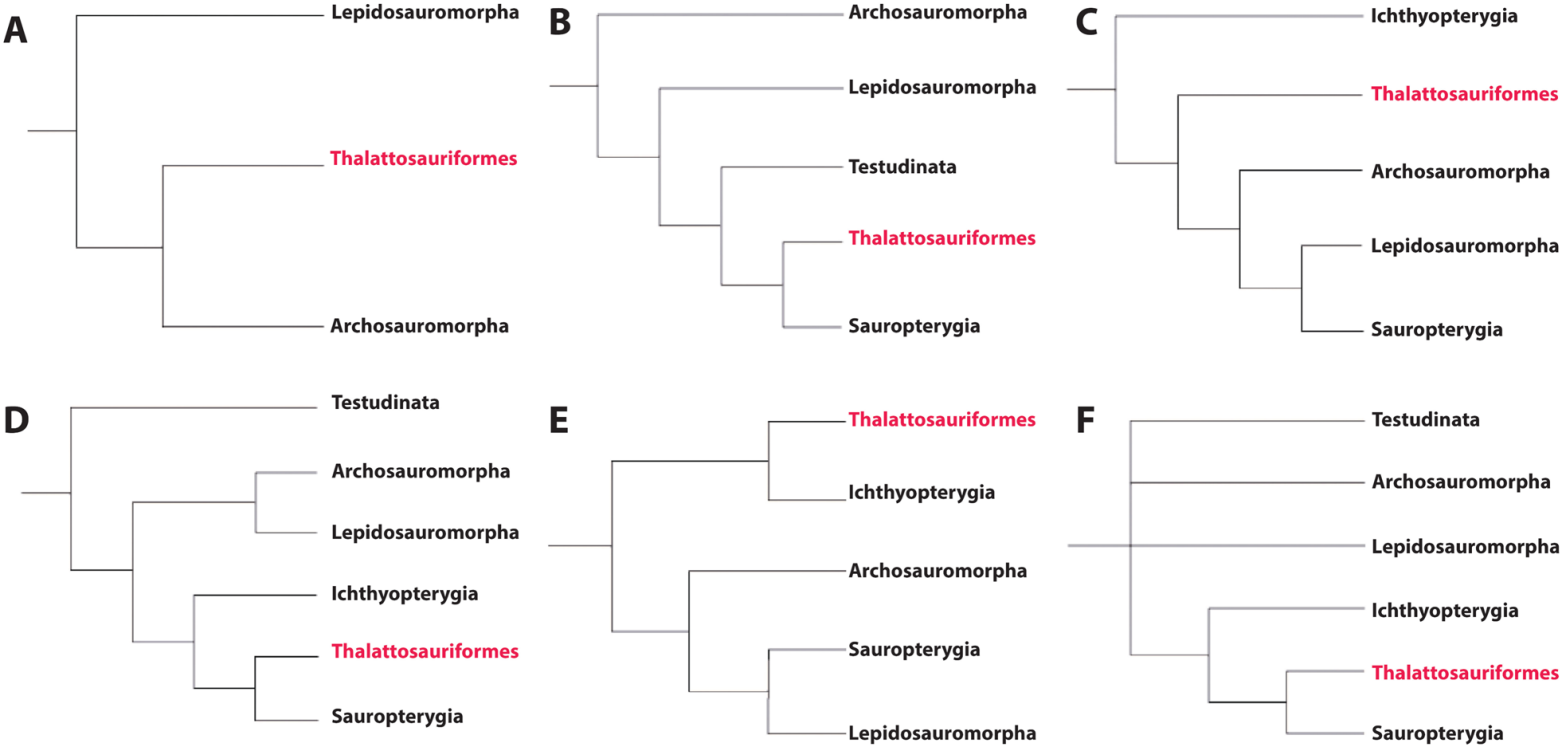
Simplified phylogenetic hypotheses proposed for the relationships of Thalattosauriformes to other reptile groups. **A** based on Evans (1988); **B** Rieppel (1998); **C** Müller (2004); **D** Neenan et al (2013); **E** Motani et al. (2015); **F** Scheyer et al (2017). Figure modified from Sun et al (2020)

**Fig. 5:**
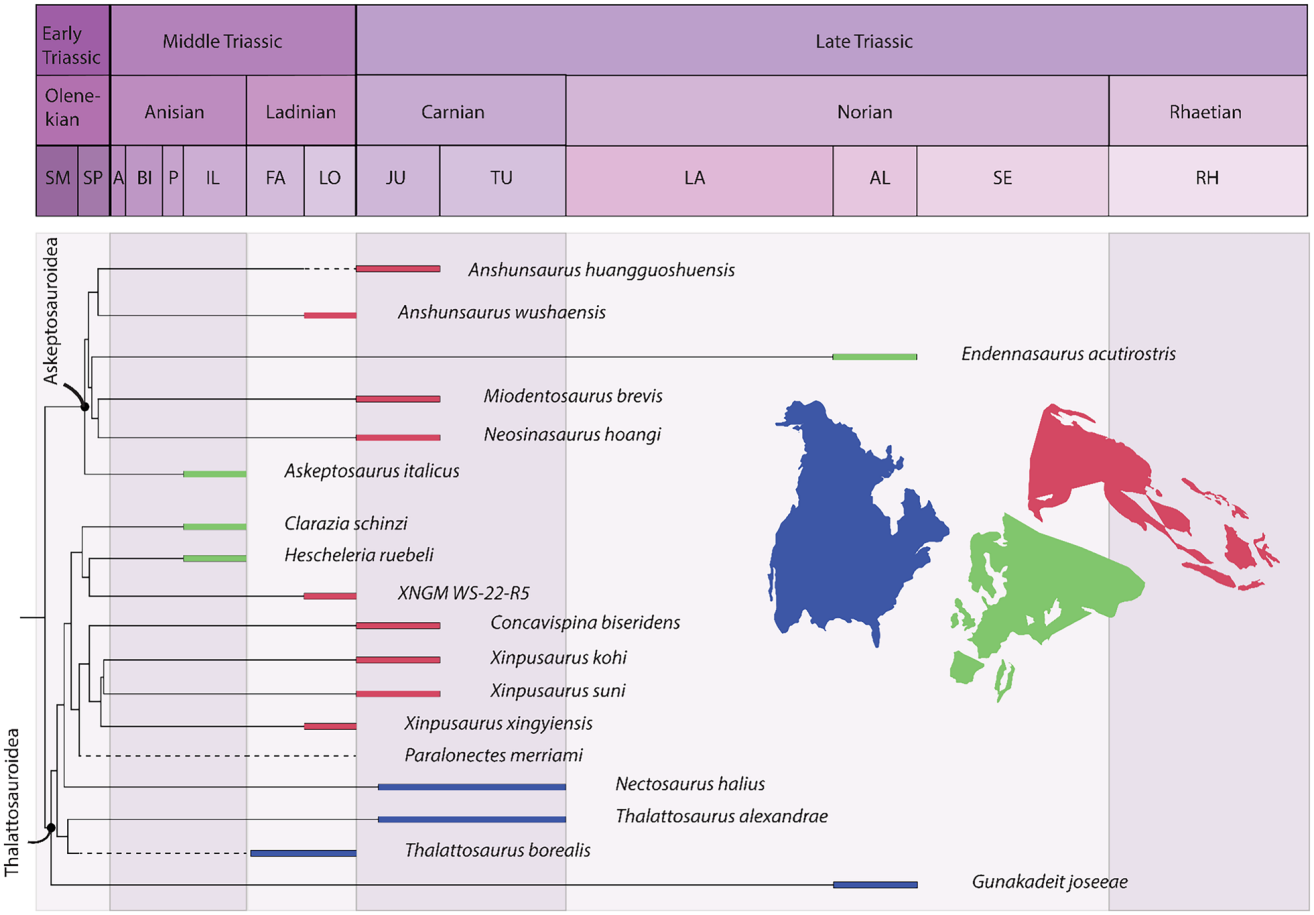
The most up-to-date time-calibrated strict consensus tree showing the interrelationships within Thalattosauriformes. The color-coded lines represent the temporal ranges of the taxa; the dashed line reflects the uncertain age ranges. The color codes reflect the spatial paleo-distribution of the clades: red represents the Eastern Tethyan Province (southwestern China), green represents the Western Tethyan Province (Europe), and blue represents the eastern Panthalassan Province (the Pacific coastline of North America). Figure modified from Jiang et al (2023), using new temporal data of *Anshunsaurus* cf. *huangguoshuensis* from Chai et al. (2020b). *SM* Smithian, *SP* Spathian, *A* Aegean, *BI* Bithynian, *P* Pelsonian, *IL* Illyrian, *FA* Fassanian, *LO* Longobardian, *JU* Julian, *TU* Tuvalian, *LA* Lacian, *AL* Alaunian, *SE* Sevetian, *RH* Rhaetian

**Fig. 6:**
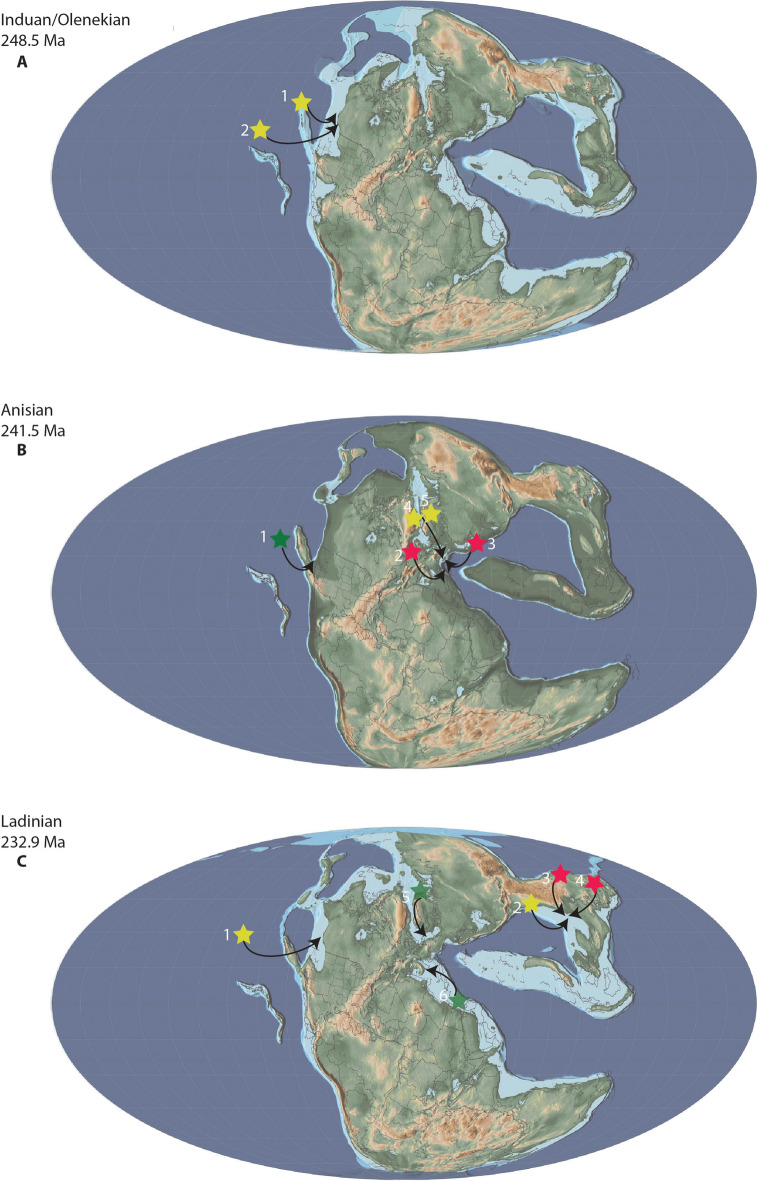
Spatiotemporal distribution of thalattosaur localities and species during the Early and Middle Triassic. Yellow stars indicate thalattosauroid localities, red stars indicate askeptosauroid localities, green indicate indeterminate thalattosaurid remains. Transparent green indicates postulated indeterminate thalattosaurid fossils. Numbers indicate different localities. Modified from Scotese (2016).** A** Induan/Olenekian, 248.5 Ma (note these are putative Lower Triassic in age). (1) Meosin Mountain, B.C.; Meosin/Sulphur Mountain Formation, Smithian, *Paralonectes* sp., *Agkistrognathus/Paralonectes* sp., Thalattosauridae indet. (RTMP collections); (2) Wapiti Lake, British Columbia; Sulphur Mountain Formation, Early Triassic, *Paralonectes* sp., *Wapitisaurus problematicus* (TMP 86.153.14), Thalattosauridae indet. (RTMP collections) (Bastiaans et al., 2023; Nicholls & Brinkman, 1993). **B** Anisian, 241.5 Ma. (1) Augusta Mountains, Nevada; Favret Formation, Anisian, Thalattosauridae indet. (Sander et al., 1994). (2) Varese, Italy; middle Besano Formation, Anisian, *Askeptosaurus italicus* (Nopcsa, 1925; Kuhn, 1952; Kuhn-Schnyder, 1960, 1971; Müller, 2005). (3) Ticino, Switzerland; middle Besano Formation, Anisian, *Askeptosaurus italicus* (Kuhn, 1952; Kuhn-Schnyder, 1960, 1971; Müller, 2005; Nopcsa, 1925). (4) Val Porina, Ticino, Switzerland; middle Besano Formation, Anisian, *Clarazia schinzi* (Peyer, 1936a; Rieppel, 1987). (5) Val Porina, Ticino, Switzerland; middle Besano Formation, Anisian, *Hescheleria ruebeli* (Peyer, 1936b; Rieppel, 1987). **C** Ladinian, 232.9 Ma. (1) Wapiti Lake, British Columbia; Sulphur Mountain Formation, upper Anisian – early Ladinian and Ladinian, *Agkistrognathus* *campbelli*, *Paralonectes merriami*, *Thalattosaurus borealis*, Thalattosauridae indet (Nicholls & Brinkman, 1993; RTMP collections). (2) Xingyi, Guizhou Province, China; Falang Formation, (upper) Ladinian, *Xinpusaurus xingyiensis*, XNGM WS-22-R5 (Chai et al., 2020a; Li et al., 2016). (3) Luoping County, Yunnan Province, China; Gjiu Formation, upper Ladinian, cf. *Askeptosaurus* (Sun et al., 2005). (4) Guizhou Province, China; Falang Formation, (upper) Ladinian, *Anshunsaurus huangnihensis, A. wushaensis, Anshunsaurus* cf. *huangguoshuensis* (Rieppel et al., 2006; Cheng, 2007a, b, 2011; Chai et al., 2020b). (5) Germany; Upper Muschelkalk, Ladinian, *Blezingeria ichthyospondylus* (Fraas, 1896; Müller, 2002; Diedrich, 2015; Schoch, 2015. (6) Tarragona, Spain; Spanish Muschelkalk, Ladinian, Potential tail fragment thalattosaur (Rieppel & Hagdorn, 1998)

**Fig. 7:**
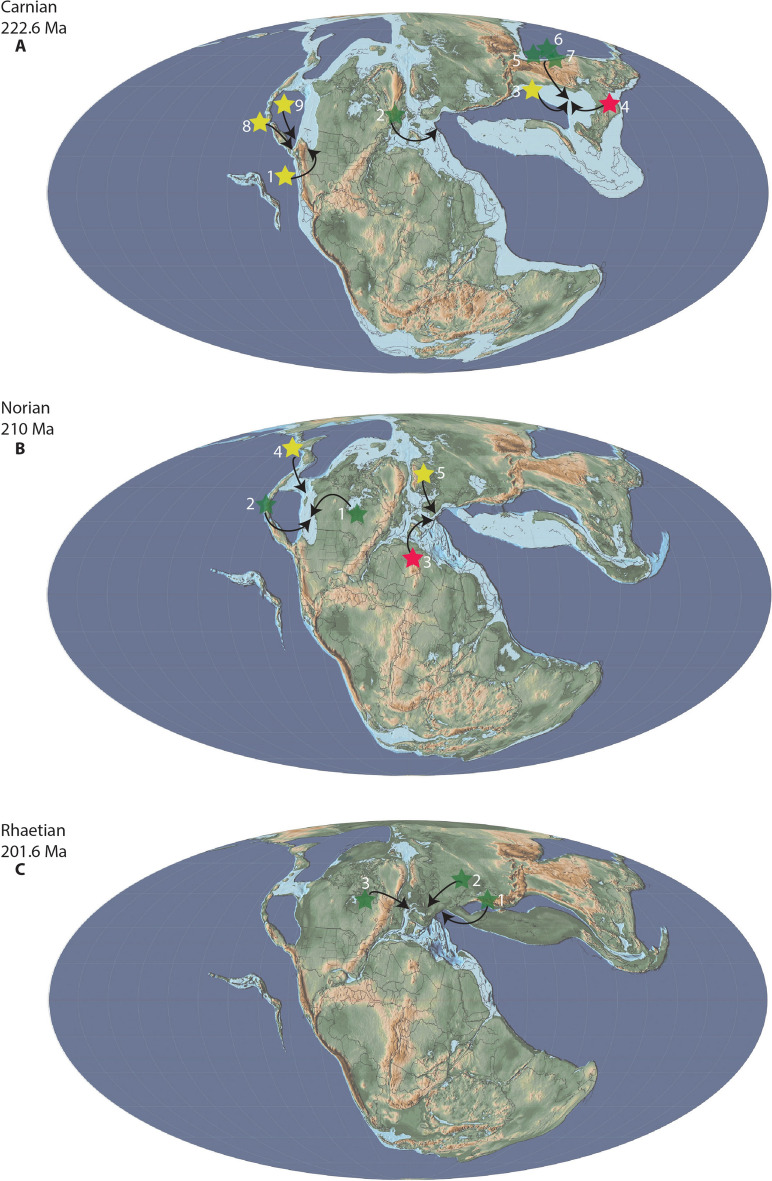
Spatiotemporal distribution of thalattosaur localities and species during the Late Triassic. Yellow stars indicate thalattosauroid localities, red stars indicate askeptosauroid localities, green indicate indeterminate thalattosaurid remains. Transparent green indicates postulated indeterminate thalattosaurid fossils. Numbers indicate different localities. Modified from Scotese (2016).** A** Carnian, 222.6 Ma. (1) Humboldt County, Nevada, USA.; Natchez Pass Formation, lower Carnian, Claraziidae indet. (USNM collections; Storrs, 1991b; Sues & Clark, 2005); (2) Julian Alps, Italy; Predil Limestone Formation, lower Carnian, potential “*Endennasaurus*-like” anterior caudals. (Dalla Vecchia, 1993). (3) Guanling County, Guizhou Province, China; Xiaowa Formation, lower Carnian, *Xinpusaurus suni, X. kohi, X. bamaolinensis, Concavispina biseridens* (Cheng, 2003; He et al., 2023; Jiang et al., 2004; Liu et al., 2013; Maisch, 2014; Rieppel & Liu, 2006; Yin et al., 2000; Zhao et al., 2013). (4) Guanling County, Guizhou Province, China; Xiaowa Formation, lower Carnian, *Anshunsaurus huangguoshuensis; Miodentosaurus *brevis (Cheng et al., 2007a, 2007b; Liu, 1999; Rieppel et al., 2000; Wu et al., 2009; Zhao et al., 2010). (5) Guanling County, Guizhou Province, China; Xiaowa Formation, lower Carnian, *Neosinasaurus hoangi* (Chai & Jiang, 2021; Yin et al., 2000). (6) Guanling County, Guizhou Province, China; Xiaowa Formation, lower Carnian, *Wayaosaurus geei* (Chai & Jiang, 2021; Yin et al., 2000). (7) Guanling County, Guizhou Province, China; Xiaowa Formation, lower Carnian, *Wayaosaurus bellus* (Chai & Jiang, 2021; Yin et al., 2000). (8) Shasta County, California, USA; Hosselkus Limestone Formation, Carnian, *Nectosaurus halius, Nectosaurus* sp., *Thalattosaurus alexandrae, T. perrini* (Merriam, 1895, 1902, 1905, 1908; Nicholls, 1999). (9) Paulina, Oregon, USA; Vester Formation, upper Carnian, Brisbois Member thalattosaur (Metz, 2019).** B** Norian, 210 Ma. (1) Pink Mountain, British Columbia, Canada; Pardonet Formation, Norian, isolated skeletal remains Thalattosauridae indet. (Storrs, 1991b). (2) Williston Lake, Jewitt Spur, Hudson's Hope, British Columbia, Canada; Pardonet Formation, Norian, cranial and postcranial remains (incl. articulated material) (ROM collections). (3) Zogno, Bergamo, Italy; Zorzino Limestone Formation, Norian, *Endennasaurus *acutirostris (Müller et al., 2005; Paganoni & Pandolfi, 1989; Renesto, 1984, 2005). (4) Keku Islands, southeast Alaska, USA; Hound Island Volcanics, middle Norian, *Gunakadeit joseeae*, Claraziidae indet. (Adams, 2009; Druckenmiller et al., 2020). (5) Gaissau, near Salzburg, Austria; Kössen Formation, upper Norian-lower Rhaetian, indeterminate thalattosauroid (Müller, 2002, 2007).** C** Rhaetian, 201.6 Ma. (1) Úbočka near Čičmany, Strážovské Mountains (Slovakia); Fatra Formation, Rhaetian, potential “*Edennasaurus*-like” femur (Čerňanský et al., 2020). (2) Baden-Württemberg, Germany; Rhaetian, *Pachystropheus rhaeticus* (Cawthorne et al., 2024; Čerňanský et al., 2020; Huene, 1935; Quinn et al., 2024; Renesto, 2005; Renesto and Dalla Vecchia, 2017; Storrs, 1992, 1994; Storrs & Gower, 1993; Storrs et al., 1996). (3) Somerset, England; Rhaetian, Fissure Fills, *Pachystropheus rhaeticus* (Cawthorne et al., 2024; Čerňanský et al., 2020; Moreau et al., 2021; Huene, 1935; Renesto, 2005; Renesto and Dalla Vecchia, 2017); Storrs, 1992, 1994; Storrs & Gower, 1993; Storrs et al., 1996 Comprehensive overview of confirmed and postulated thalattosauriform-bearing formations and information on repositories containing thalattosaurian material University of Oregon Museum of Natural and Cultural History, Eugene, Oregon, USA Simplified phylogenetic hypotheses proposed for the relationships of Thalattosauriformes to other reptile groups. **A** based on Evans (1988); **B** Rieppel (1998); **C** Müller (2004); **D** Neenan et al (2013); **E** Motani et al. (2015); **F** Scheyer et al (2017). Figure modified from Sun et al (2020) The most up-to-date time-calibrated strict consensus tree showing the interrelationships within Thalattosauriformes. The color-coded lines represent the temporal ranges of the taxa; the dashed line reflects the uncertain age ranges. The color codes reflect the spatial paleo-distribution of the clades: red represents the Eastern Tethyan Province (southwestern China), green represents the Western Tethyan Province (Europe), and blue represents the eastern Panthalassan Province (the Pacific coastline of North America). Figure modified from Jiang et al (2023), using new temporal data of *Anshunsaurus* cf. *huangguoshuensis* from Chai et al. (2020b). *SM* Smithian, *SP* Spathian, *A* Aegean, *BI* Bithynian, *P* Pelsonian, *IL* Illyrian, *FA* Fassanian, *LO* Longobardian, *JU* Julian, *TU* Tuvalian, *LA* Lacian, *AL* Alaunian, *SE* Sevetian, *RH* Rhaetian Spatiotemporal distribution of thalattosaur localities and species during the Early and Middle Triassic. Yellow stars indicate thalattosauroid localities, red stars indicate askeptosauroid localities, green indicate indeterminate thalattosaurid remains. Transparent green indicates postulated indeterminate thalattosaurid fossils. Numbers indicate different localities. Modified from Scotese (2016).** A** Induan/Olenekian, 248.5 Ma (note these are putative Lower Triassic in age). (1) Meosin Mountain, B.C.; Meosin/Sulphur Mountain Formation, Smithian, *Paralonectes* sp., *Agkistrognathus/Paralonectes* sp., Thalattosauridae indet. (RTMP collections); (2) Wapiti Lake, British Columbia; Sulphur Mountain Formation, Early Triassic, *Paralonectes* sp., *Wapitisaurus problematicus* (TMP 86.153.14), Thalattosauridae indet. (RTMP collections) (Bastiaans et al., 2023; Nicholls & Brinkman, 1993). **B** Anisian, 241.5 Ma. (1) Augusta Mountains, Nevada; Favret Formation, Anisian, Thalattosauridae indet. (Sander et al., 1994). (2) Varese, Italy; middle Besano Formation, Anisian, *Askeptosaurus italicus* (Nopcsa, 1925; Kuhn, 1952; Kuhn-Schnyder, 1960, 1971; Müller, 2005). (3) Ticino, Switzerland; middle Besano Formation, Anisian, *Askeptosaurus italicus* (Kuhn, 1952; Kuhn-Schnyder, 1960, 1971; Müller, 2005; Nopcsa, 1925). (4) Val Porina, Ticino, Switzerland; middle Besano Formation, Anisian, *Clarazia schinzi* (Peyer, 1936a; Rieppel, 1987). (5) Val Porina, Ticino, Switzerland; middle Besano Formation, Anisian, *Hescheleria ruebeli* (Peyer, 1936b; Rieppel, 1987). **C** Ladinian, 232.9 Ma. (1) Wapiti Lake, British Columbia; Sulphur Mountain Formation, upper Anisian – early Ladinian and Ladinian, *Agkistrognathus* *campbelli*, *Paralonectes merriami*, *Thalattosaurus borealis*, Thalattosauridae indet (Nicholls & Brinkman, 1993; RTMP collections). (2) Xingyi, Guizhou Province, China; Falang Formation, (upper) Ladinian, *Xinpusaurus xingyiensis*, XNGM WS-22-R5 (Chai et al., 2020a; Li et al., 2016). (3) Luoping County, Yunnan Province, China; Gjiu Formation, upper Ladinian, cf. *Askeptosaurus* (Sun et al., 2005). (4) Guizhou Province, China; Falang Formation, (upper) Ladinian, *Anshunsaurus huangnihensis, A. wushaensis, Anshunsaurus* cf. *huangguoshuensis* (Rieppel et al., 2006; Cheng, 2007a, b, 2011; Chai et al., 2020b). (5) Germany; Upper Muschelkalk, Ladinian, *Blezingeria ichthyospondylus* (Fraas, 1896; Müller, 2002; Diedrich, 2015; Schoch, 2015. (6) Tarragona, Spain; Spanish Muschelkalk, Ladinian, Potential tail fragment thalattosaur (Rieppel & Hagdorn, 1998) Spatiotemporal distribution of thalattosaur localities and species during the Late Triassic. Yellow stars indicate thalattosauroid localities, red stars indicate askeptosauroid localities, green indicate indeterminate thalattosaurid remains. Transparent green indicates postulated indeterminate thalattosaurid fossils. Numbers indicate different localities. Modified from Scotese (2016).** A** Carnian, 222.6 Ma. (1) Humboldt County, Nevada, USA.; Natchez Pass Formation, lower Carnian, Claraziidae indet. (USNM collections; Storrs, 1991b; Sues & Clark, 2005); (2) Julian Alps, Italy; Predil Limestone Formation, lower Carnian, potential “*Endennasaurus*-like” anterior caudals. (Dalla Vecchia, 1993). (3) Guanling County, Guizhou Province, China; Xiaowa Formation, lower Carnian, *Xinpusaurus suni, X. kohi, X. bamaolinensis, Concavispina biseridens* (Cheng, 2003; He et al., 2023; Jiang et al., 2004; Liu et al., 2013; Maisch, 2014; Rieppel & Liu, 2006; Yin et al., 2000; Zhao et al., 2013). (4) Guanling County, Guizhou Province, China; Xiaowa Formation, lower Carnian, *Anshunsaurus huangguoshuensis; Miodentosaurus *brevis (Cheng et al., 2007a, 2007b; Liu, 1999; Rieppel et al., 2000; Wu et al., 2009; Zhao et al., 2010). (5) Guanling County, Guizhou Province, China; Xiaowa Formation, lower Carnian, *Neosinasaurus hoangi* (Chai & Jiang, 2021; Yin et al., 2000). (6) Guanling County, Guizhou Province, China; Xiaowa Formation, lower Carnian, *Wayaosaurus geei* (Chai & Jiang, 2021; Yin et al., 2000). (7) Guanling County, Guizhou Province, China; Xiaowa Formation, lower Carnian, *Wayaosaurus bellus* (Chai & Jiang, 2021; Yin et al., 2000). (8) Shasta County, California, USA; Hosselkus Limestone Formation, Carnian, *Nectosaurus halius, Nectosaurus* sp., *Thalattosaurus alexandrae, T. perrini* (Merriam, 1895, 1902, 1905, 1908; Nicholls, 1999). (9) Paulina, Oregon, USA; Vester Formation, upper Carnian, Brisbois Member thalattosaur (Metz, 2019).** B** Norian, 210 Ma. (1) Pink Mountain, British Columbia, Canada; Pardonet Formation, Norian, isolated skeletal remains Thalattosauridae indet. (Storrs, 1991b). (2) Williston Lake, Jewitt Spur, Hudson's Hope, British Columbia, Canada; Pardonet Formation, Norian, cranial and postcranial remains (incl. articulated material) (ROM collections). (3) Zogno, Bergamo, Italy; Zorzino Limestone Formation, Norian, *Endennasaurus *acutirostris (Müller et al., 2005; Paganoni & Pandolfi, 1989; Renesto, 1984, 2005). (4) Keku Islands, southeast Alaska, USA; Hound Island Volcanics, middle Norian, *Gunakadeit joseeae*, Claraziidae indet. (Adams, 2009; Druckenmiller et al., 2020). (5) Gaissau, near Salzburg, Austria; Kössen Formation, upper Norian-lower Rhaetian, indeterminate thalattosauroid (Müller, 2002, 2007).** C** Rhaetian, 201.6 Ma. (1) Úbočka near Čičmany, Strážovské Mountains (Slovakia); Fatra Formation, Rhaetian, potential “*Edennasaurus*-like” femur (Čerňanský et al., 2020). (2) Baden-Württemberg, Germany; Rhaetian, *Pachystropheus rhaeticus* (Cawthorne et al., 2024; Čerňanský et al., 2020; Huene, 1935; Quinn et al., 2024; Renesto, 2005; Renesto and Dalla Vecchia, 2017; Storrs, 1992, 1994; Storrs & Gower, 1993; Storrs et al., 1996). (3) Somerset, England; Rhaetian, Fissure Fills, *Pachystropheus rhaeticus* (Cawthorne et al., 2024; Čerňanský et al., 2020; Moreau et al., 2021; Huene, 1935; Renesto, 2005; Renesto and Dalla Vecchia, 2017); Storrs, 1992, 1994; Storrs & Gower, 1993; Storrs et al., 1996

1 and in the captions of Figs. 1, 2, 3, 4, 5, 6, 7. Personal observations mention collection identification numbers and institute.

## Abbreviations

IVPP: Institute of Vertebrate Paleontology and Paleoanthropology, Beijing, China
MBSN: Museo Civico di Scienze Naturali 'E. Caffi', Bergamo, Lombardy, Italy
MSNM: Museo di Scienze Naturali Milano, Italy
PIMUZ: Paleontological Institute of the University of Zurich, Zurich, Switzerland
(R)TMP: (Royal) Tyrrell Museum of Palaeontology, Drumheller, Alberta, Canada
UCMP: University of California, Museum of Paleontology, Berkeley, California, USA
USNM: United States National Museum, Smithsonian Institution, Washington DC, USA
XNGM: Xingyi National Geopark Museum, Xingyi, Guizhou Province, China
ZMNH: Zhejiang Museum of Natural History, Hangzhou, Zhejiang, China
